# Supplementary figures and images for: Computational and robotic modeling reveal parsimonious combinations of interactions between individuals in schooling fish
Source: PLoS Comput Biol. 2020 Mar 16;16(3):e1007194. doi: 10.1371/journal.pcbi.1007194 (PMC7098660; doi:10.1371/journal.pcbi.1007194)

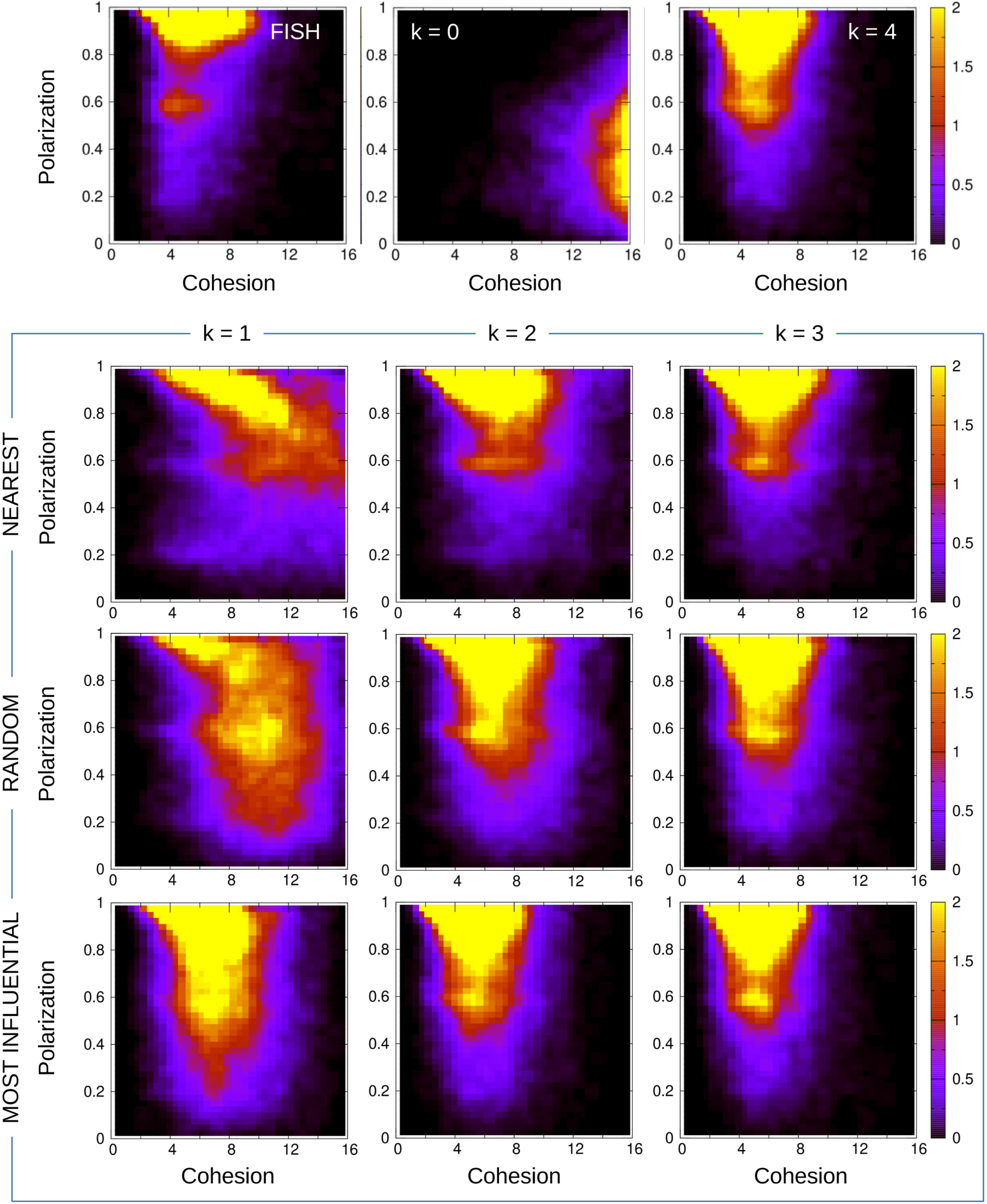

Supplement: S1 Fig — Density maps are shown for fish experiments (fish panel) and for the 11 strategies considered in the model simulations. The color intensity corresponds to the number of data in each box normalized with the total number of data in the grid (×1000). We used 40 × 50 boxes. (TIF) [file pcbi.1007194.s001.tif]

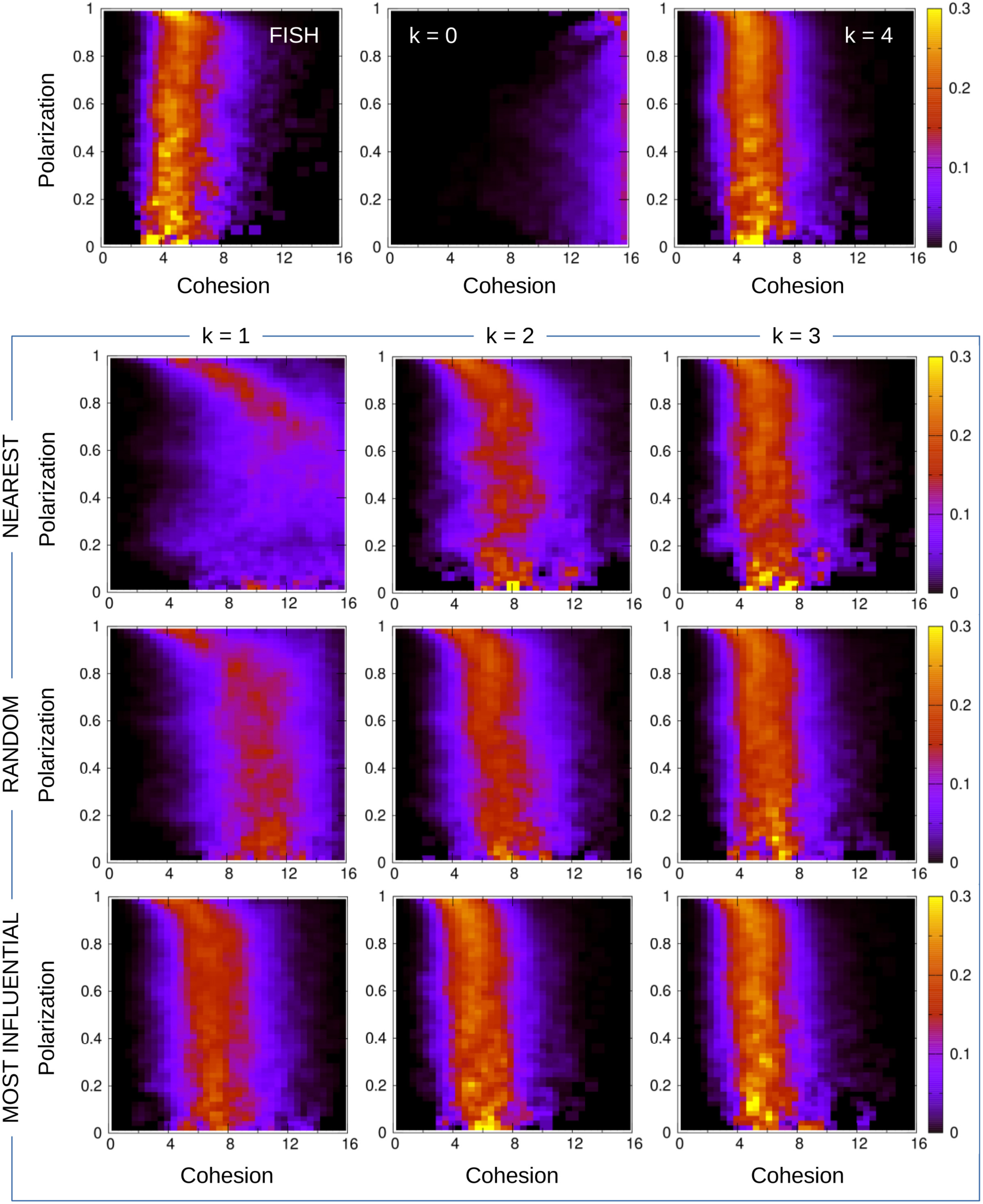

Supplement: S2 Fig — Density maps are shown for fish experiments (fish panel) and for the 11 strategies considered in the model simulations. The color intensity corresponds to the number of data in each box normalized with the number of data per interval of polarization, i.e., each row is the PDF of the cohesion for a range of values of the polarization. We used 40 × 50 boxes. (TIF) [file pcbi.1007194.s002.tif]

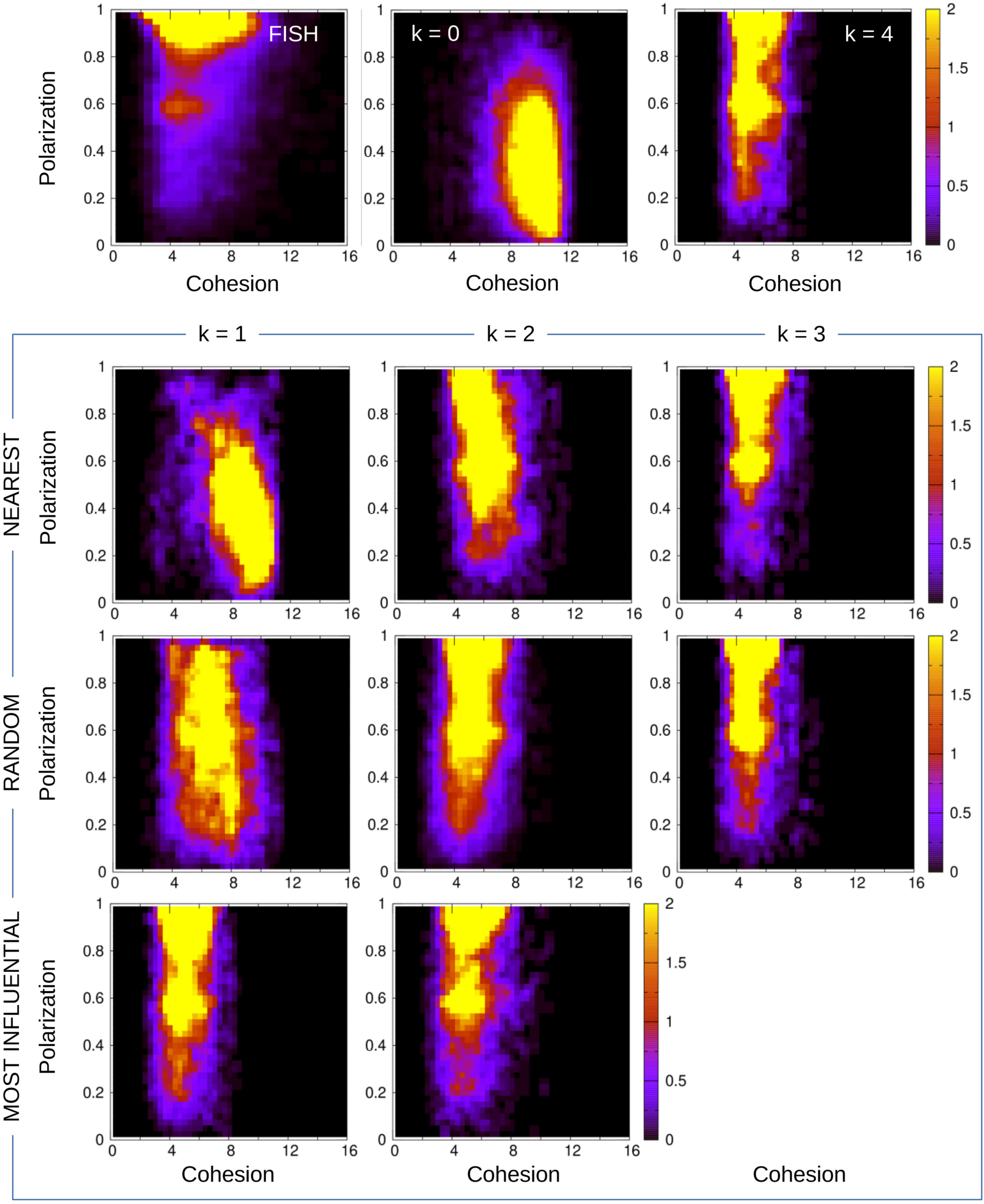

Supplement: S3 Fig — Density maps are shown for fish experiments (fish panel) and for the 10 strategies considered in the robot experiments. The color intensity corresponds to the number of data in each box normalized with the total number of data in the grid (×1000). We used 40 × 50 boxes. (TIF) [file pcbi.1007194.s003.tif]

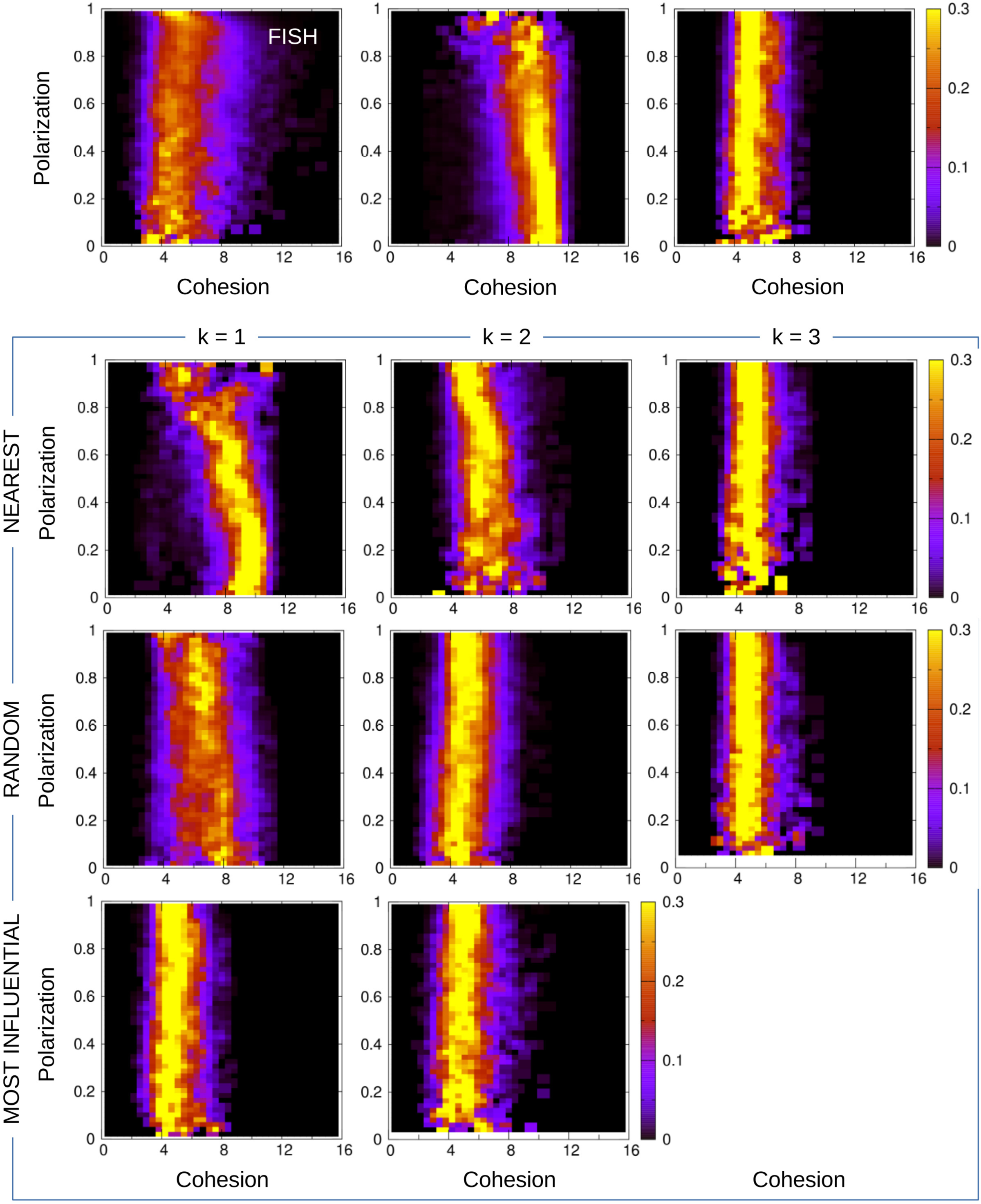

Supplement: S4 Fig — Density maps are shown for fish experiments (fish panel) and for the 10 strategies considered in the robot experiments. The color intensity corresponds to the number of data in each box normalized with the number of data per interval of polarization, i.e., each row is the PDF of the cohesion for a range of values of the polarization. We used 40 × 50 boxes. (TIF) [file pcbi.1007194.s004.tif]

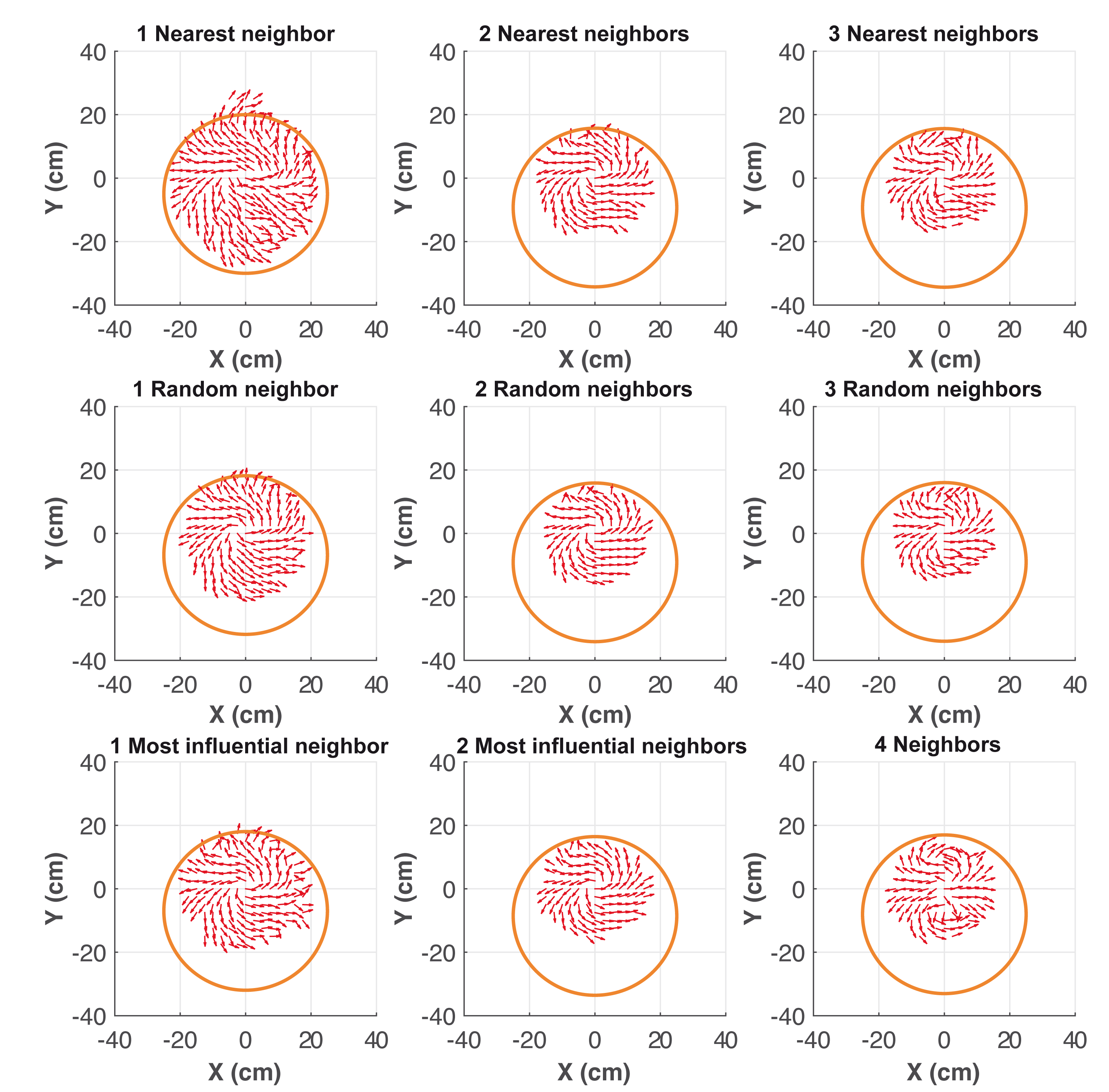

Supplement: S5 Fig — Red arrows represent the velocity field of agents in the reference system of the barycenter of the group, here located at coordinates (0, 0). Orange circle denotes the average relative position of the border of the arena with respect to the barycenter. The cases where agents interact with the k = 3 most influential neighbors are statistically identical to the case where k = 4. (TIF) [file pcbi.1007194.s005.tif]

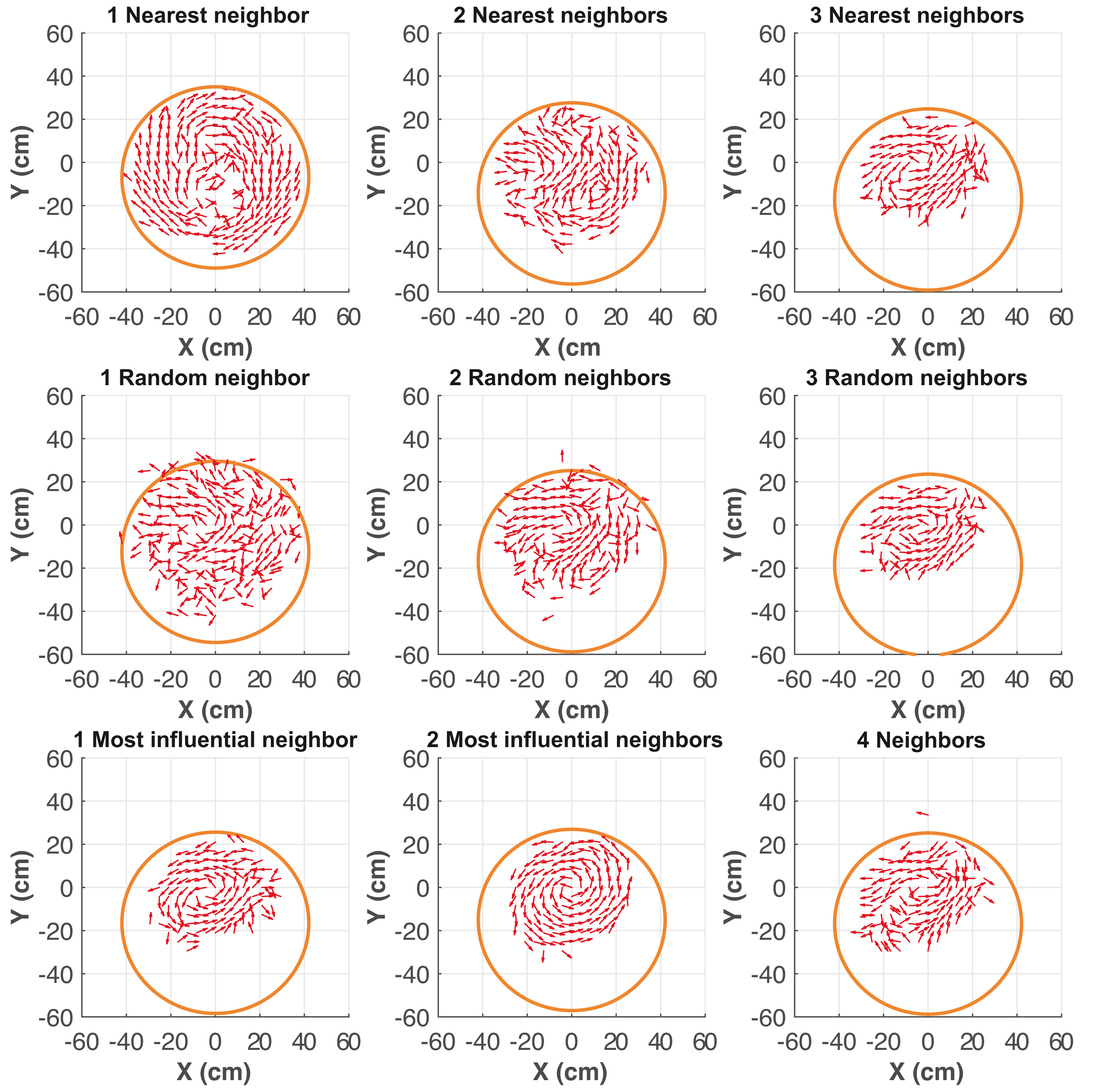

Supplement: S6 Fig — Red arrows represent the velocity field of robots in the reference system of the barycenter of the group, here located at coordinates (0, 0). Orange circle denotes the average relative position of the border of the arena with respect to the barycenter. The cases where robots interact with the k = 3 most influential neighbors are statistically identical to the case where k = 4. (TIF) [file pcbi.1007194.s006.tif]

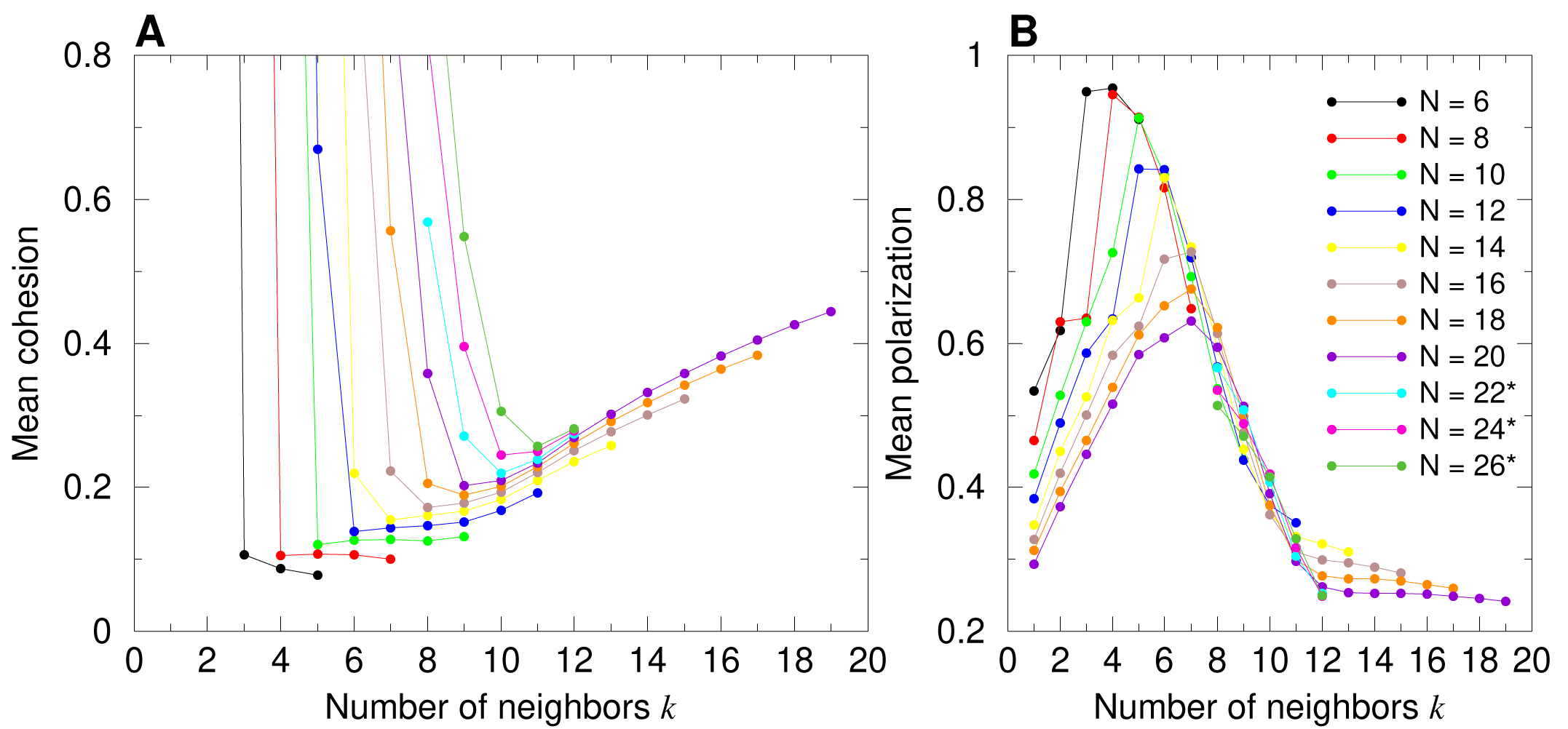

Supplement: S7 Fig — Mean cohesion (A) and mean polarization (B) as a function of k. Cohesion values are scaled with λM = 0.87. In panel (A), high values of the cohesion for small values of k with respect to the group size N grow up to 20 m in our simulations as the individuals diffuse independently of each other (vertical lines). In (B), the values of k for N = 22, 24 and 26 (marked with an asterisk in the legend) are limited to the interval of interest [8, 12]. (TIF) [file pcbi.1007194.s007.tif]

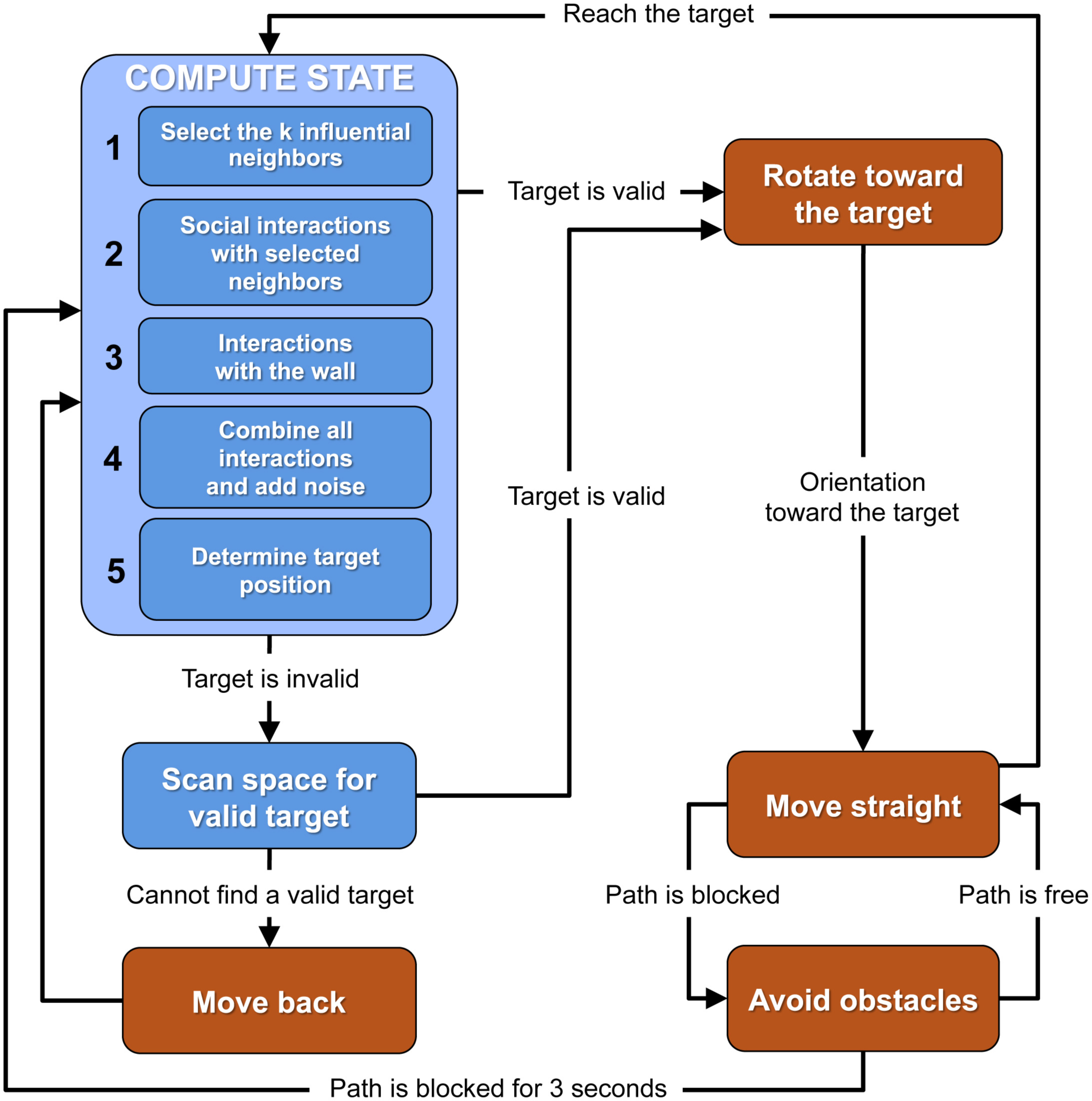

Supplement: S8 Fig — The decision-making processes of the robot (COMPUTE state) are shown in blue. The movements of the robot (MOVE state) are shown in brown. In the COMPUTE state, the model determines a new target to reach by integrating the local information about the neighbors and the environment. A target is valid when this one is not blocked by the wall or other robots. If the target is invalid, the computer tries to find a new target by the scanning method. If the scanning fails, the robot moves back 80 mm and starts again for model computing. If the decision target is valid, the robot switches into MOVE state, which includes three sub-states: Rotate, Move straight, and Avoid obstacle. The robot first rotates towards to the target and then moves straight to it. If a running neighbor blocks the path, the robot uses a procedure to avoid the obstacle. (TIF) [file pcbi.1007194.s008.tif]

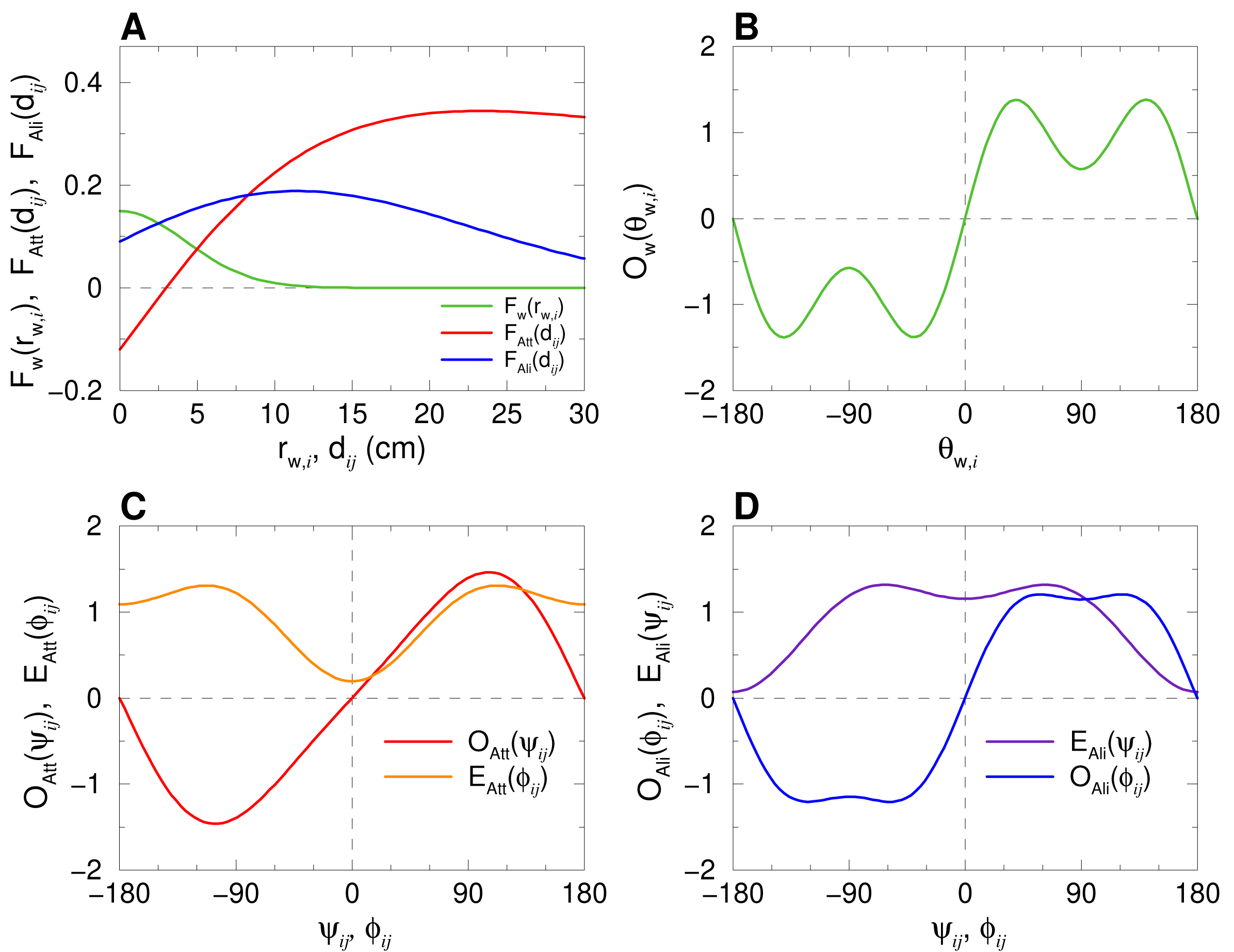

Supplement: S9 Fig — (A) Intensity of the repulsion from the wall Fw(rw,i) (green) as a function of the distance to the wall rw,i, and intensity of the attraction FAtt(dij) (red) and the alignment FAli(dij) (blue) between fish i and j as functions of the distance dij separating them. (B) Normalized odd angular function Ow(θw,i) modulating the interaction with the wall as a function of the relative angle to the wall θw,i. (C) Normalized angular functions OAtt(ψij) (odd, in red) and EAtt(ϕij) (even, in orange) of the attraction interaction, and (D) OAli(ϕij (odd, in blue) and EAli(ψij) (even, in violet) of the alignment interaction between agents i and j, as functions of the angle of perception ψij and the relative heading ϕij. (TIF) [file pcbi.1007194.s009.tif]
