# Supplementary material for: Computational and robotic modeling reveal parsimonious combinations of interactions between individuals in schooling fish
Source: PLoS Comput Biol. 2020 Mar 16;16(3):e1007194. doi: 10.1371/journal.pcbi.1007194 (PMC7098660; doi:10.1371/journal.pcbi.1007194)
Supplement: Attachment — Submitted filename: Rebutal_Letter.pdf [file pcbi.1007194.s024.pdf]

Dear Editor,

Please find the revised version of our paper *"Computational and robotic modeling reveal parsimonious combinations of interactions between individuals in schooling fish"*.

We thank the reviewers for their careful reading of the manuscript and their constructive remarks. We have taken the comments on board to improve and clarify the manuscript, and in particular the three points that have been underlined by the Editor. Please find below a summary of the main changes in the new version of the manuscript and our detailed point-by-point response to all reviewers' comments. We have also included a 'track changes' document (attached as a supplement to our response to the Editor).

Sincerely,

Guy Theraulaz et al.

*The paper requires major revisions in order to address all the comments made by the reviewers, which are valid and should be carefully considered. Special attention should be paid to the following:*

*1. Although the "most influential neighbour" hypothesis can explain the observations from experiments, it is not fully justified from a cognitive point of view (one reviewer talks about 'self-referential' definition, another one about the computational load required to individuals to identify the most-influential neighbour), and neither the computational nor the robotic model seems to address this point.*

*2. There is a lack of quantitative comparison between the results obtained with different models. This is particularly important to support the authors' claims.*

*3. The contribution obtained from implementing the model in a (seemingly centralised) robotic system is not clear, and should be better explained*

**Before specifically addressing the comments of the 3 reviewers, we list below the main changes in the new version of the manuscript in relation to the above editorial summary. We are confident that these changes also resolve the reviewers' main criticisms.**

- **a)** The introduction to the Results section has been substantially extended and improved in order to address several points raised by the 3 reviewers (fish swimming mode and "kicks"; origin, "data-driven" nature, and ingredients of the model; relevant integrated variables by the fish; strategies to select the interacting neighbors; definition of the most influential neighbors and its biological interpretation; quantifiers of the "similarity" between fish experiments and model/robots…).
- **b)** In particular, the notion of most influential neighbors is now much better explained and justified (starting in the introduction of the Results section), and its biological relevance is also addressed in the final discussion (which now recapitulates more concisely the main results of our study).

**c)** We have added a brief introduction to the section devoted to robot experiments in the Result section (where our motivations for using a robotic platform are also addressed; see also a slight change in the abstract), and the discussion of these experiments and their motivations in Materials and Methods has been extended and improved (see also Fig. 12-14).

**d)** The Results section has been drastically improved (style, clarity, precision, notations…). In particular, it has been made much more precise by the introduction of the Hellinger distance between two measured probability distributions in order to quantify their (dis)similarity (see the new last section of Material and Methods). The new Table 1 and 2 in the text list these distances for the three interaction strategies considered in this work compared to fish experiments.

**e)** We have extended our simulations for larger groups of model agents moving in an unbounded domain (a more thorough study will be addressed in the future). See Fig. 9 and the new Fig. 10 and S7.

**f)** We have revised and extended the general discussion in order to underline the main contribution of our results in the wider context of attention and visual information processing in fish

## Answers to the 3 reviewers' comments:

### Reviewer #1:

*In the paper "Computational and robotic modeling reveal parsimonious combinations of interactions between individuals in schooling fish" the authors study collective motion in fish by means of mathematical modeling and using swarm robots as models. The main focus is on how many neighbors need to be taken into consideration for an individual in order to generate a valid collective motion with appropriate cohesion and polarization.*

*The subject is relevant and within scope. The paper is overall well written and relatively easy to follow. Several logical entities are not explained and discussed in one place but are rather distributed over the whole paper. As a consequence, a few aspects are difficult to understand, especially for a reader new to collective motion in fish (details below). Another problem is that the overall contribution of the paper is a bit difficult to assess although the finding of small neighbor selections being effective is relevant. A third issue is that neither the robot experiments themselves nor the methodology of the robot experiments are sufficiently described.*

***#1.1.*** *A - distributed and incomplete explanations*

*The description of the robot methodology is distributed over at least two sections, imprecise, and incomplete.*

**A:** We thank the Reviewer for pointing this out. We have modified the robot methodology as follows: We first introduce the main structure of our robotic platform in Fig. 12. Then we have modified Fig. 13 to better illustrate the difference between the numerical simulations done with the SPP model and the control mechanism used in the Hardware in loop simulation. The main difference is that the robot simulation needs two extra function

modules (motion control & obstacle avoidance) to ensure safe movement. So, we added more details on these two modules to make our robot simulation more clearly.

In order make the robot methodology more coherent, we moved the description of the **"Implementation of the behavioral model in the robot"** in the section describing the "**Swarm robotic platform**" and we added more explanations in the section "**Experimental platform**". We have thus amended the revised manuscript to explain more fully the difference between robot methodology and the software simulation:

***Experimental platform***

*Fig. 13 (red and blue boxes) shows the "hardware in loop" (HIL) simulation used to control the Cuboids robots. The HIL simulation integrates the robots hardware into the distributed control loops of the platform computer software. As such, it differs from a traditional software simulation, being a semi-real one. Compared with pure theoretical simulations "in silico" (i.e. the software simulation box in Fig.13), the HIL simulation integrates both the hardware constraints (i.e. the mechanical constraints of the robots, the time delay of the control loop which includes the shooting by the camera, the time of calculation and sending orders by the WIFI router) and those that result from the movement of the robots in a physical environment, in particular the need to avoid collision with obstacles and other robots (see the blue box in Fig. 13). The main difference between the HIL simulation and the software simulation is the real time control of the behavior of each robot, which is achieved by the Motion Control and Real Time Control modules (see the red box in Fig. 13).*

*The Motion Control mechanism includes two motion patterns: the first one is a spot rotation, which means the robot rotates around its center by means of wheels differential driving. The speed control of the two wheels is described by the following equation:*

$$V_{Ri} = -V_{Li} = p_t \delta\phi_{ci}, \qquad (1)$$

*where* $V_{Ri}$, $V_{Li}$ *are the speeds of right and left wheels of the robot and* $p_t$ *a factor of proportionality with a constant value.* $\delta\phi_{ci}$ *is the real time value of heading variation which is determined by the Real Time Control module. The other motion pattern of the robot is Moving straight. When this happens, the speeds of the left and right wheel are the same and:*

$$V_{Ri} = V_{Li} = p_m l_{ci}, \qquad (2)$$

*where* $p_m$ *is a factor of proportionality with a constant value and* $l_{ci}$ *is the value of kick length which is also determined by the Real Time Control module.*

*The function of Real Time Control module is ensuring the safe movement of robots. This module first converts the decision of the Computational Model* $[\delta\phi_i(t_{dec}), l_i(t_{dec})]$ *at the last decision time* $t_{dec}$ *into a real time decision that is then performed by the robot* $[\delta\phi_{ci}(t), l_{ci}(t)], t > t_{dec}$. *The Algorithm below provides the details of Real Time Control module.*

Algorithm of the Real Time Control module

**Input:**

Computational Model decision $[\delta\phi_i(t_{\text{dec}}), l_i(t_{\text{dec}})]$. real time position $[x_{ci}(t), y_{ci}(t), \phi_{ci}(t)]$

**Output:**

real time decision $[\delta\phi_{ci}(t), l_{ci}(t)]$

$\delta\phi_{ci}(t) = \phi_i(t_{\text{dec}}) + \delta\phi_i(t_{\text{dec}}) - \phi_{ci}(t), t > t_{\text{dec}}$

$l_{ci}(t) = l_i(t_{\text{dec}}) - \sqrt{(x_{ci}(t) - x_i(t_{\text{dec}}))^2 + (y_{ci}(t) - y_i(t_{\text{dec}}))^2}$

**If** $|\delta\phi_{ci}(t)| > \delta\phi_{\text{Threshold}}$

  **Spot rotation** (1) for Motion Control in real time.

**Else**

  **Moving straightly** (2) for Motion Control in real time.

**If** the target is reached ( $|\delta\phi_{ci}(t)| < \delta\phi_{\text{Threshold}} \,\&\, l_{ci}(t) < l_{\text{Threshold}}$ )?

  **Goto COMPUTE** state (computational model) for a new decision.

**If** the path is not free

  do Obstacle Avoidance procedure``.

**End**

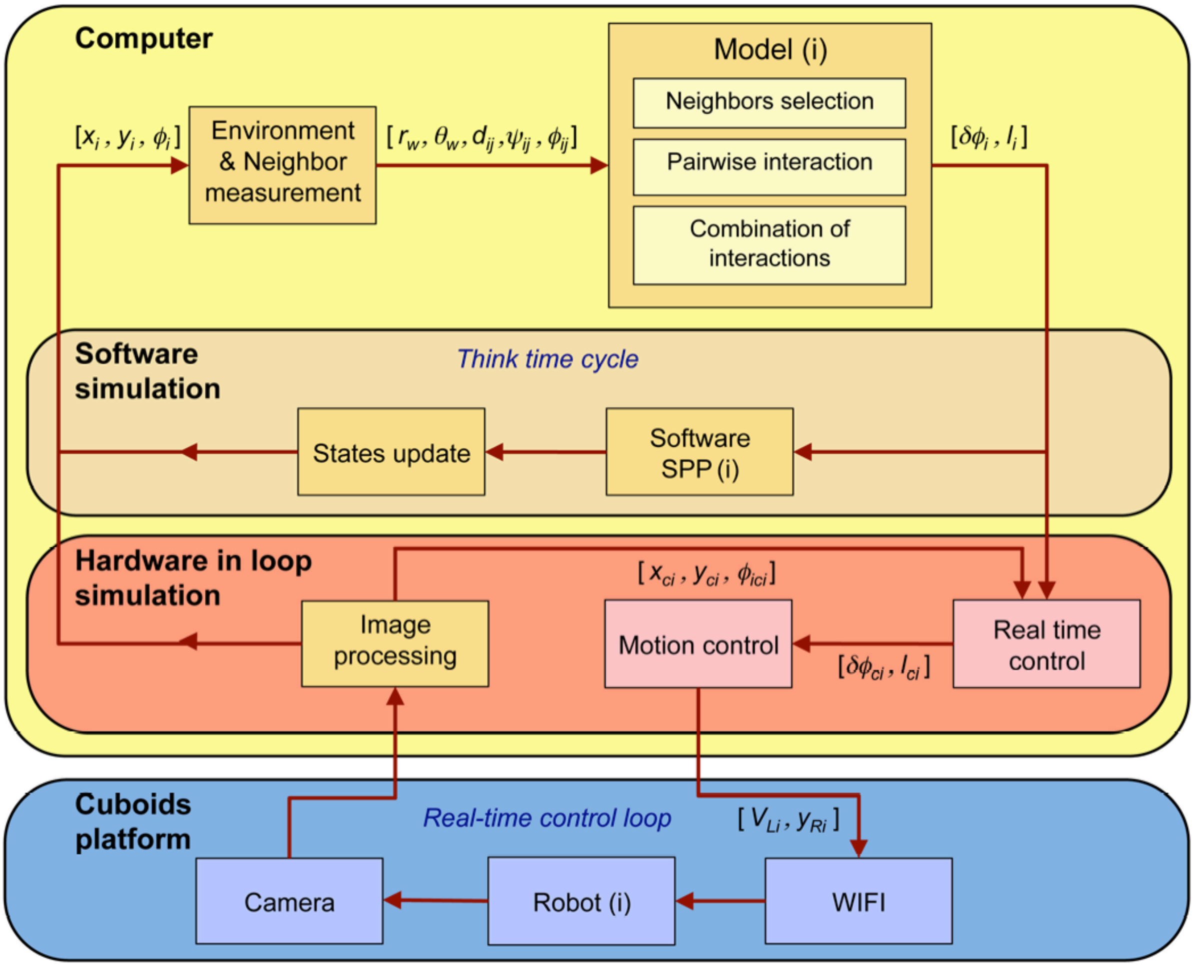

**Fig 13. Software simulation and Hardware in Loop (HIL) simulation.** The structure of HIL is an extension of the software simulation, which consists of two extra parts: 1) a computer software (Motion Control, Real Time Control and Image Processing modules) and 2) a physical hardware (Robot, camera and wireless router). In the software simulation, the Environment & Neighbor Measurement module converts the global position of a robot or a Particle in the SPP software $[x_i, y_i, \phi_i]$ into local information $[\theta_{\mathrm{W}i}, r_{\mathrm{W}i}]$, $[d_{ij}, \psi_{ij}, \Delta\phi_{ij}]$. Then the computational model generates a new kick decision as the form of heading variation and kick length $[\delta\phi_i, l_i]$. This new decision $[\delta\phi_i, l_i]$ is then directly sent to the SPP($i$) software. Once the state has been updated, a new global position is provided by the SPP($i$) software (brown box) or the Hardware in loop simulation (red box). By contrast, the HIL simulation includes hardware, i.e. robots, camera and WIFI router, (blue box). Furthermore, each robot ($i$) is controlled in real time by three more software modules running in the computer, which are the *Image Processing*, *Motion Control* and *Real Time Control* modules (red box). The Image Processing module computes the global position of each robot $[x_{\mathrm{c}i}, y_{\mathrm{c}i}, \phi_{\mathrm{c}i}]$ from the information provided by the camera in real time. Then, the Real Time Control module converts the model decision $[\delta\phi_i, l_i]$ into a real time decision in the robot $[\delta\phi_{\mathrm{c}i}, l_{\mathrm{c}i}]$, which are the heading variation and kick length to perform the decision based its real time position $[x_{\mathrm{c}i}, y_{\mathrm{c}i}, \phi_{\mathrm{c}i}]$. Finally, the Motion Control software generates left and right wheel motors speed control $[V_{\mathrm{L}i}, V_{\mathrm{R}i}]$ for each robot to achieve its decision $[\delta\phi_{\mathrm{c}i}, l_{\mathrm{c}i}]$. Each robot receives these motor commands by WIFI signals, and performs the corresponding movements that are monitored by the camera.

***Implementation of the behavioral model in the robots (moved after the experimental platform section)***

*We use LabVIEW object-oriented programming (OOP) tool to design the distributed control software for the Cuboids robots (Fig. 13). It first establishes independent memories for each robot as an agent to store real time information, such as robot ID, location and heading* $[x_{\mathrm{c}i}(t), y_{\mathrm{c}i}(t), \phi_{\mathrm{c}i}(t)]$ *at time step* $t$, *and real time decision* $[\delta\phi_{\mathrm{c}i}(t), l_{\mathrm{c}i}(t)]$. *We design a state machine control structure to implement the HIL simulation control for each robot. With the new speed control command determined by the Motion Control module, the actuators of the robot are controlled wirelessly by the WIFI signals sent by the computer. The robot controls its wheels to move towards the new target place while LED colors display the state of the robot.*

*Robots use a constant kick length* $l_i(t_{\mathrm{dec}})$ *of around 8 cm, that is, twice the body length of a robot, which corresponds to the mean kick length measured in experiments with five fish. Using a constant straight step also allows to check if the new target place can be reached or not, in particular, to prevent the case where the agent could be intercepted by another agent, in which case the distance traveled by the agent will be shorter than* $l_i(t_{\mathrm{dec}})$.

*The state machine control structure for an individual robot includes two main states: COMPUTE state and MOVE state (Fig. 14). The robots are programmed to perform a burst-and-coast movement mimicking the swimming mode of fish. When a robot is in the*

*COMPUTE state at $t_{\mathrm{dec}}$, the computational model determines a new decision $[\delta\phi_i(t_{\mathrm{dec}}), l_i(t_{\mathrm{dec}})]$. After that, the robot switches to the MOVE state and adjusts its wheels to move towards the decision place in real time thanks to the Motion Control and Real Time Control modules. Since other robots are moving around asynchronously, the robot must avoid these dynamic obstacles while being in the MOVE state. To prevent collisions between robots, we designed and implemented an obstacle avoidance protocol. When no valid targets can be generated during the COMPUTE state (due to the impediment imposed by nearby robots), the robot generates a valid target place by means of a scanning method and, alternatively, just moves back over a short distance. However, this circumstance rarely occurs in our experiments.*

*We describe below the two states and the additional procedures used to avoid collisions with dynamical obstacles.*

- *COMPUTE State: This state generates a new decision $[\delta\phi_i(t_{\mathrm{dec}}), l_i(t_{\mathrm{dec}})]$ for the focal robot by means of the computational model, which is programmed in MATLAB. In this state, the robot takes the information about its local environment $[\theta_{\mathrm{W}i}, r_{\mathrm{W}i}]$ and selects the neighbors to be taken into account corresponding to the current local interaction strategy. Then the robot computes the variation of its heading angle $\delta\phi_i(t_{\mathrm{dec}})$ that combined with the kick length $l_i(t_{\mathrm{dec}})$ to determine a new target place. The location of new target is then checked and validated by the OOP software so as to avoid any collision with static obstacles, before the robot switches to the MOVE state (see Fig. 14). While a robot is in the COMPUTE State, the white LED light is turned on.*

- *MOVE State: In this state the robot evaluates whether its heading angle $\phi_{\mathrm{c}i}$ is aligned with the new place target. If the deviation $\delta\phi_{\mathrm{c}i}$ is too large, the robot first rotates towards the target and then moves straight until it reaches the target thanks to the Motion Control module. Then, when the robot successfully reaches the target, it returns to the COMPUTE state to determine a new target. While a robot is in the MOVE State, the green LED light is turned on.*

- *Obstacle Avoidance Protocol: This procedure is triggered as soon as the target path of the focal robot $i$ crosses the safety zone of another robot $j$. The safety zone is a circular area around a robot with diameter of 80 mm. In this case, the focal robot $i$ first stops and computes whether it can continue moving or not according to the information it has about the distance $d_{ij}$ and relative angular position $\psi_{ij}$ of the neighboring robot. If the focal robot has the moving priority (determined by a large value of the angle of perception, $\psi_{ij} > 90^\circ$, meaning that the robot is a temporary leader), or if the distance $d_{ij}$ is larger than the diameter of the circle of security ($d_{ij} > 80$ mm, meaning that the robot $j$ is far enough away), the moving condition is satisfied and the focal robot $i$ successfully switches back into the MOVE state. If not, it repeatedly checks the values $d_{ij}$ and $\psi_{ij}$ with the neighboring robot until the moving condition is satisfied. If the focal robot cannot go back into the MOVE state within 3 seconds, it toggles to the COMPUTE state to determine a new target.*

- *No Valid Target Procedure: This procedure is triggered when the robot is in the COMPUTE state and cannot generate a valid target place within 3 seconds. In this situation, the robot scans the local environment from its front to the nearest neighbor located at one of its sides.*

*If there exists a free space for generating a target place, the robot toggles to the MOVE state. If, after scanning, no free space is available for moving, the robot moves back over a predefined distance of 80 mm (approximately two robot body lengths) and then toggles to the COMPUTE state to determine a new target place.*

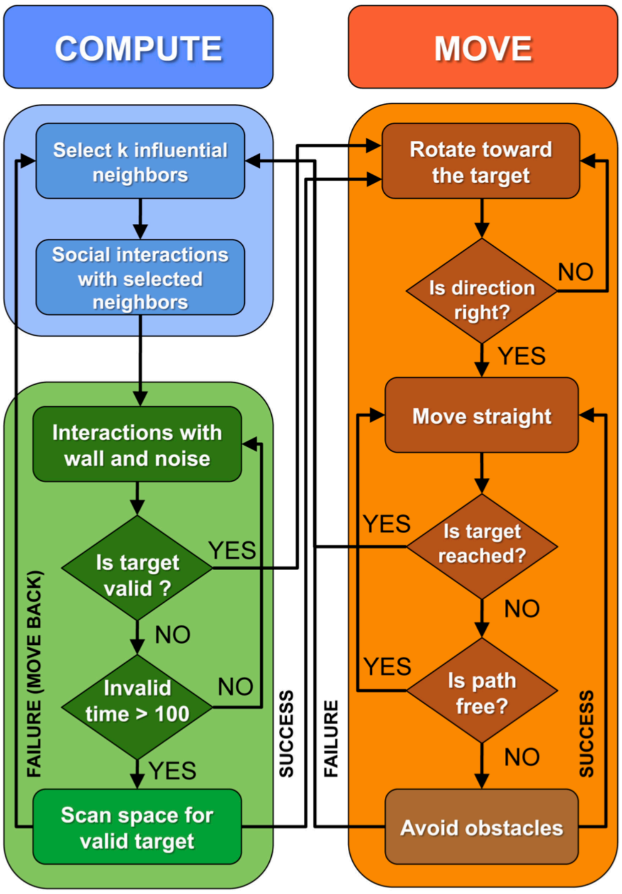


**Fig 14. Flow chart of robot states machine.** At any time a robot can be in one of the two following states: (1) the COMPUTE state for choosing a new target place, and (2) the MOVE state to reach the target place. In the COMPUTE state, the robot first selects influential neighbors, then it computes the pairwise influence of each neighbor, and finally it adds all influences to generate a new target place. Then, this new target place is validated to avoid collisions with the wall or another robot. If a valid target place cannot be found, the robot scans all space around itself for a valid target place. If the scanning method cannot find a valid target, the robot moves back over a distance of 80mm and starts again the COMPUTE state. When a valid target place has been found, the robot switches into the MOVE state. The robot first rotates towards to the target and then, moves straight to it. If another running neighbor blocks the path, the robot uses a procedure to avoid the obstacles.

**#1.2.** *A crucial term is that of "kick" which is frequently used before it is vaguely defined. Please introduce the term before using it and try to define it as accurate as possible. Since you are using an automated kick detection to process the biological data, it may make sense*

*to report that in connection to that definition. Similarly, "kick length" should be defined more clearly - possibly as displacement or covered distance.*

**A:** We have significantly extended and improved the introduction of the Results section. In particular, the burst-and-coast swimming mode is described from the beginning, including the definition of a "kick" (the model description in Materials and Methods has also been improved to make the notion of kick clearer).

**#1.3.** *The description of most figures is distributed over the whole results section. If a reader wants to look up the text for a given figure, then that is basically impossible.*

**A:** In our work, we analyzed the impact of 3 different interaction strategies on the collective behavior both in numerical simulations and robotic experiments. In order to make the reading of our results easier and to avoid too many graphs, we chose to group the results on the simulations and robots for each of the 6 measures (5 in the revised version) that we used to quantify collective movements. To further improve the synthetic presentation of results and as suggested by Reviewers 2 and 3, we have introduced the Hellinger distance to quantify the similarity between the theoretical distributions obtained in model simulations and robotic experiments with those obtained in our experimental results with fish. These synthetic results are presented in Tables 1 and 2 (presented after the main figures, following the PLOS requirements for the submitted manuscript).

**#1.3.** *B contribution*

*It is a bit difficult to assess the significance of the contribution. There is a contribution and it is relevant but more discussion is required. How does this work relate to your own previous publications and what are the most closely related papers of other groups? The introduction is great to read and gives a good overview of related work but those papers are then not directly compared to this paper's contribution.*

**A:** Very few works have explicitly addressed the question about how individuals combine and integrate interactions with their neighbors in a group of moving animals. In the introduction we have indicated the very few theoretical works related to this question. Moreover, to the best of our knowledge, no other works has rigorously extracted and model the functional forms of social interactions between pairs of individuals in animal groups. As a consequence, it was quite difficult to investigate in a rigorous way the combination and integration of such pairwise interactions by individuals and their impact on collective motion. Still, previous works have used different methods at least to identify influential neighbors in birds and fish. We have extended the discussion to include these previous works.

*"Collective motion involving the coherent movements of groups of individuals is primarily a coordination problem. Each individual within a group must precisely adjust its behavior to that of its neighbors in order to produce coordinated motion. Determining how these relevant neighbors are chosen at the individual scale is therefore a key element to understand the coordination mechanisms in moving animal groups.*

*Previous experimental works on fish and birds have identified interacting neighbors using short-term directional correlations (Vicsek & Zafeiris, 2012; Nagy et al., 2010) or anisotropy of the position of the nearest neighbors (Ballerini et al., 2008). In a starling flocks (Sturnus vulgaris), each bird coordinates its motion with a fixed number of closest neighbors (six to seven), irrespective of their distance (Ballerini et al., 2008). However in fish schools, experimental studies suggest that each individual only interacts with a lower number of influential neighbors. In the mosquitofish (Gambusia holbrooki) each fish mostly interacts with one single nearest neighbor (Herbert-Read et al., 2011). In the rummy nose tetra (Hemigrammus rhodostomus) during collective U-turns, the analyses of directional correlations between fish suggest that each fish mainly reacts to one or two neighbors at a time. These results are in line with theoretical works that have suggested that, instead of averaging the contributions of a large number of neighbors, as suggested by many models (Aoki, 1982; Huth & Wissel, 1992; Niwa, 1994; Vicsek et al., 1995; Couzin et al., 2002; Gautrais et al., 2012), individuals could pay attention to only a small number of neighbors [Lemasson et al., 2009; Bode et al., 2010; Lemasson et al., 2013; Calvao and Brigatti, 2014). This mechanism would overcome the natural limitation of amount of information each individual can handle (Dukas et al., 2002).”*

References cited:


Vicsek T. and Zafeiris A. (2012) Collective motion. *Physics Reports*. 2012; 517(3): 71-140.

Nagy M, Akos Z, Biro D and Vicsek T (2010) Hierarchical group dynamics in pigeon flocks. *Nature* 464: 890–893.

Ballerini M, et al. (2008) Interaction ruling animal collective behavior depends on topological rather than metric distance: Evidence from a field study. *Proc Natl Acad Sci USA* 105: 1232–1237.

Herbert-Read JE, Perna A, Mann, RP, Schaerf, TM, Sumpter, DJ and Ward, AJ. (2011) Inferring the rules of interaction of shoaling fish. *Proc Natl Acad Sci USA*. 2011; 108(46): 18726-18731.

Aoki I (1982). A simulation study on the schooling mechanism in fish. *Bull. J. Soc. Sci. Fish* 48(8): 1081-1088.

Huth A and Wissel C. (1992) The simulation of the movement of fish schools. *J. Theor. Biol.* 156(3): 365-385.

Niwa HS. (1994) Self-organizing dynamic model of fish schooling. *J. Theor. Biol.* 171(2): 123-136

Couzin, ID, Krause, J, James, R, Ruxton, G and Franks, NR (2002) Collective memory and spatial sorting in animal groups. *J. Theor. Biol.* 218(5): 1-11.

Vicsek, T, Czirok, A, Ben-Jacob, E, Cohen, I and Shochet, O (1995). Novel Type of Phase Transition in a System of Self-Driven Particles. *Phys. Rev. Lett.* 75: 226{1229.

J. Gautrais et al. (2012). Deciphering interactions in moving animal groups. *PLoS Comp. Biol.* 8(9): e1002678.

Lemasson, BH, Anderson, JJ and Goodwin RA (2009). Collective motion in animal groups from a neurobiological perspective: the adaptive benefits of dynamic sensory loads and selective attention. *J. Theor. Biol.* 261(4):501-510.

Bode NW, Franks DW and Wood AJ. (2011) Limited interactions in flocks: relating model simulations to empirical data. *J. R. Soc. Interface* 8, 301–304

Lemasson, BH, Anderson, JJ and Goodwin RA (2013). Motion-guided attention promotes adaptive communication during social navigation. *Proc. R. Soc. B*: 280(1754): 20122003.

Calvao, AM and Brigatti, E (2014). The Role of Neighbours Selection on Cohesion and Order of Swarms. *PLoS ONE* 9(5): e94221.

Dukas, R (2002). Behavioural and ecological consequences of limited attention. *Phil. Trans. R. Soc. Lond. B* 357:1539{1547.

**#1.4.** C - *robot experiments*

*It seems likely to me that the robots are completely controlled centrally and externally. It seems that the robot uses none of its own sensors. However, that remains crucially unclear because it is mentioned that the robots are "perfectly autonomous" and (only?) the decision-making is externalized. The use or non-use of sensors is not discussed. A better description of Fig. 12 within the text may help here. It remains also unclear whether or how frequent kicks of the robots are enforced given that robots need to turn on spot often. How do you, for example, prevent that a robot just keeps turning for long as the requirements for to where turn change while it is turning?*

**A:** We thank the referee for pointing out this issue. In our experiments, the robots do not use their own sensors because the goal was to test the computational model with real physical constrains. The decisions of the robots are entirely controlled by the main computer. At each decision time and for each robot, the computer generates a decision $[\delta\phi_i, l_i]$ according the outcome of the computational model (see the new version of Fig. 13). Then, in the move MOVE state, the computer controls the behavior of each robot in real time until it reaches the target safely. So due to the real time supervision of top camera, the main computer knows the position and orientation of all robots, which help the computer to control the robot turning or moving. To improve the clarity we have modified Fig.13.

We have improved the section describing the experimental platform to clarify the aforementioned points.

*"Although the robot has its own sensors to ensure it autonomous control and movements, in this work, we used a remote-control mode. This is because our goal was to compare the performances between the software simulation and the robot experiment with the same computational model and the same local information input (see the Hardware In Loop simulation in Fig. 13). There exists a real time control loop that ensures the safe movement of each robot and which help a robot to rotate towards the new target place and move straight to the target place (see the red and blue boxes in Fig. 13)."*

**#1.5.** *There is basically no text about how the robot experiments were done. The platform and setup are described but not how many experiments under what conditions were executed for how long etc.*

**A:** We agree with the referee that more information is needed. We added the following sentences in the section describing the experimental platform (the section has also been globally improved):

*“The setup was placed in a chamber made by 3 opaque wooden boards and 1 white curtain. 2 LED light panels provide a diffused lighting. A circular cardboard wall of radius R=420 mm delimited the border of experimental platform. The floor of the experimental platform was made with a rough wooden board that prevented the reflections of light.”*

We also added the following sentences and information in the section describing local interactions strategies:

*“With the computational model, for each combination of interactions, we performed 50 simulations for a total of 8 × 104 kicks for all the 5 agents (about 21 hours per condition).*

*In the robotic experiments, we performed one experiment for each combination of interactions with about 8000 kicks in average for all the 5 robots. The duration time of experiments performed for each condition was the following: no interactions between robots (150 min), interacting only with k = 1 nearest neighbor (61 min), interacting only with k = 2 nearest neighbors (62 min), interacting only with k = 3 nearest neighbors (63 min), interacting only with k = 1 randomly chosen neighbor (65 min), interacting only with k = 2 randomly chosen neighbors (128 min), interacting only with k = 3 randomly chosen neighbors (48 min), interacting only with k = 1 most influential neighbor (68 min), interacting only with k = 2 most influential neighbors (82 min), interacting with k = 4 neighbors (150 min)”*

**#1.6.** *The necessity of the robot experiments is not well motivated, even more so if the robot control is centralized and robots are only remotely controlled. What are specific embodied features that you can test in this robot setup?*

**A:** The motivation to perform the robot experiments is twofold and mainly concerns the impact of collision avoidance and the physical constraints on collective movements (the consequences in the case k=1 for the NEAREST strategy are indeed major). On one hand, traditional software simulation uses a Self-Propelled Particle model to represent the behavior of the agents (fish) and their interactions. Since an agent is always treated as a point, the strict avoidance behavior between particles is not included (note that fish in shallow water can also cross each other, due to the 3D nature of the actual experimental setup). Moreover, a robot can only be considered as a rigid body. So when many robots get together, they have to keep enough space around them to prevent collision and move coherently. Thus, a first goal of the robotic experiments was to test the robustness of the different interaction strategies between the robots when avoidance constraints are taken into account. To manage the collision avoidance constraints, we added two extra software modules, a *Real Time Control* module and a *Motion Control* module that are combined to the computational model. On the other hand, there exist non-holonomic constraints in a mobile robot. This causes a robot to take more time to turn in spot than a particle in the SPP model. Moreover, there exist many sources of physical noise, such as mechanical friction, electrical disturbance and time delay of the control loop whose impact on the interactions is unknown.

The request made by the referee is similar to the one above (# 1.1). We have added an introduction to the Results section addressing robots and extended the corresponding section in Materials and Methods in order to better explain the scientific challenges addressed by the robotic experiments and the differences with “in silico” simulations of the computational model.

**#1.7.** *It would have been easier to review, if the figures would have been embedded in the text. For a possible revision, please mark all your changes in color.*

**A:** We agree with the referee that the inclusion of figures/tables in the text allows for an easier reading of the manuscript. Unfortunately, the submission format imposed by *Plos Computational Biology* requires to regroup all figures/tables after the main text of the article. To simplify reading and corrections to the text we have included a track changes document.

Detailed comments

**#1.11.** *1 - abstract, “data-based”: To me it is unclear in what sense the model can be described as data-based.*

**A:** In [14], the repulsive interaction between a fish and the wall of the tank and the alignment/attraction interactions between fish have been measured by exploiting ~70 hours of 1-fish and 2-fish trajectories. The measured interactions were then directly implemented in the model, so that the model is not just a phenomenological model with guessed albeit reasonable interactions, hence the term “data-based”. We have made this point much clearer in the new introduction of the Results section. **Note that the introduction of this Results section has been substantially extended and improved in order to address several points raised by the 3 referees (fish swimming mode and “kicks”, origin, “data driven” nature, and ingredients of the model, relevant integrated variables by the fish, strategies to select the interacting neighbors, influential neighbors…).**

**#1.12.** *2 - abstract, “only with the neighbor having the strongest effect on its heading variation”: This can be misread as a self-referential definition. In fact, there is no better definition in the whole paper. It is only repeated twice later into the paper.*

**A:** We changed the sentence to make it clear that the effect is meant on the focal fish. Given the position and orientation of the focal fish and its neighbors, the model output is the contribution in the turning angle of the focal fish induced by each of its neighbor. The most influential neighbor is the one potentially leading to the largest absolute contribution. We have made this definition much clearer in the introduction of the Results section (see above) and now also discuss the biological relevance of the notion of most influential neighbors in the introduction of the Results section and in the final discussion.

**#1.13.** *3 - p. 3, "brings us back to a often": to _an_ often*

**A:** Changed to "an".

**#1.14.** *4 - results, six quantities: I understand that the main definition follows in the method section; still, a natural language definition in half a sentence per quantity would be useful.*

**A:** We have modified and extended the introduction of the Results section to give a more intuitive definition of these quantities, which are now presented more clearly in an itemized list. **Note that in the revised version, we only use the 5 most relevant quantities.**

**#1.15.** *5 - p. 4&5, counter-milling: From the explanation in the text one could interpret counter-milling as an artifact of the experiment setup. It what sense is it a relevant and/or natural behavior?*

**A:** It is certainly a feature of a group of fish constrained to follow a concave boundary and this phenomenon has a simple physical interpretation given at the end of the Results – "Collective behavior in fish experiments" section. Of course, in our experiment this boundary is perfectly circular, but there is no reason to believe that counter-milling is a mere artifact of the experimental setup. In any case, a faithful model for a fish school should be able to reproduce the behavior of real fish swimming along a circular boundary. We have improved the physical interpretation of counter-milling in the Materials & Methods and Results sections.

**#1.16.** *6 - p. 5, "formation of group": formation of _a_ group*

**A:** "a" added: "formation of a compact group"

**#1.17.** *7 - p. 7, Fig. S6: Figure and data are difficult to understand based on the little provided information*

**A:** Figure S6 illustrates the counter-milling phenomenon in the robotics experiments. It is similar to Figure S5 that shows the exact same information in the model simulations. The results shown on Figure S6 are discussed in the section *"Collective behavior in swarm robotics experiments"*. The counter-milling phenomenon is only clearly visible when robots interact with their k=2 most influential neighbors.

**#1.18.** *8 - p. 7, "scaling factor" lambda: The concept of the scaling factor is difficult to understand based on the given information.*

**A:** In particular for robot experiments, the spatial scales (but also, the temporal scales) are different than those in the fish experiments. Hence, to compare spatial PDF (and only those), it is necessary to translate the scales for robots into comparable distances in fish experiments. We have added the following justification in the Results section part addressing the scaling factor for robots: *"Despite the fact that the spatial and temporal scales of the robotic platform have been scaled at best to correspond to that of the fish experiments (in particular, 4x4cm square robots in an arena of radius R=42cm vs elongated fish of typical length 3cm swimming in a tank of radius R=25cm), the border and other robots have a stronger effect on a focal robot at short distance. Indeed, as explained above, the collision avoidance protocol (see Materials and Methods) induces effective interactions between the robots that have a longer range than the interactions between fish. In addition,*

*the square shape of the robot also makes them effectively bigger than if they were elongated like fish. Hence, the rescaling of distances as measured in robot experiments is necessary to be able to compare the different spatial distributions in fish and robot experiments, although it does not affect polarization, counter-milling, or angular distributions. As a result, we found a much smaller scaling factor than in model simulations: lambda_R = 0.35. Note that once the optimal scaling factor is determined, it is kept fixed in all considered situations (strategy to select the interacting neighbors and their number k). From now, all distances in the robot experiments mentioned in this section are hence expressed after rescaling to be comparable to corresponding distances in the fish experiments."*

**#1.19.** *9 - p. 10, description of "most influential neighbor": concept still unclear and seemingly self-referential*

**A:** See our reply to 1.11 and 1.12 above, and the introductory text listing the main changes in the manuscript.

**#1.20.** *10 - p. 11, "150 L": 150 l?*

**A:** Changed.

**#1.21**. *11 - p. 11, "box to isolate": unclear, what kind of box?*

**A:** We have rewritten this part and added more information about the experimental setup:

*"We used a rectangular experimental tank of size 120 X120 cm, made of glass supported by a structure of metal beam 20cm high. A plywood plate was interposed between the mesh and the basin to dampen the forces exerted on the glass basin by its own weight and water. This structure also enables the attenuation of vibrations."*

**#1.22.** *12 - p. 12, "Cuboids … they are 60 m high": seems not right*

**A:** Changed.

**#1.23.** *13 - p. 12, "messages infrared signals": please correct*

**A:** Changed; it now reads "... a robot can send messages (infrared signals) by the..."

**#1.24.** *14 - p. 12, "neighboring robot that transmit": please correct*

**A:** Changed.

**#1.25.** *15 - p. 12, "by triangulation method": please correct*

**A:** Changed; it now reads *"... calculated by a method of triangulation."*

**#1.26.** *16 - p. 13, "kind of bubble sort algorithm": This is maybe not the best comparison; also bubble sort is considered to be one of the worst sorting algorithms*

**A:** The Reviewer is right, although we are only sorting 5 elements so speed is not relevant. We however changed the text to *"the procedure is a sorting algorithm where..."*

**#1.27.** *17 - p. 14, "…is the kick length": Please give a comprehensive definition.*

**A:** We added "(*i.e.*, the distance traveled until the next kick)". We also improved the first sentence of the section. Now, kicks are also better defined in the introduction of the Results section.

**#1.28.** *18 - p. 16, "being those given in Table 1": missing parenthesis*

**A:** Changed.

**#1.29.** *19 - p. 17, "among the robot": among the _robots_*

**A:** Changed.

## Reviewer #2:

*This article contributes to the important line of research aiming at revealing the nature of cognitive and interaction mechanisms used by social animals to perform collective motion. In this work, the authors analyze collective motion data of a group of 5 H. rhodostomus fish (by computing a set of group-level metrics), and develop two synthetic models to test their understanding on the modalities of group interactions: a computational model and a swarm robotics model. The main finding of the paper is that individual fish need only to interact with one "influential" neighbor to achieve good level of coherence and order, and that interacting with two neighbors (no matter how chosen) is enough for the models to replicate closely the data of the fish.*

*This paper is an important addition to the literature and I would like to see it published. I have however a number of moderate to high level concerns that apply to the manuscript in its current form:*

**#2.1.** *In the introduction, paragraph starting at line 98, the author claim that their goal is to "better understand how individual combine and integrate interactions with their neighbors in a group of moving animals". However, the study is mainly focused on the number of neighbors the focal individual needs to interact with, not really on the mechanism by which information combing from neighbors is combined. A similar statement that needs to be slightly aligned with the results is the one at lines 109-113 (it is not clear here whether one needs one neighbors or two or three, while later it is clear that one neighbor is enough to reproduce collective motion and two-three to reproduce the data). I believe these statements in the introduction should be revised to achieve alignment between contribution statement and results.*

*I believe some essential details to assess the importance of this contribution are located in the materials and methods section, however they should briefly outlined in the main body of the paper as well. In the study of collective motion, two important questions are what type of information is exchanged between individuals (e.g. orientation only vs relative position and bearing only vs both), and how this information is integrated (see my previous point).*

**A:** In [14], the interactions between a fish and the wall of the tank and between 2 fish were quantitatively measured and then implemented in the model and the relevance of the variables involved in the model (some mentioned above) was extensively studied and

demonstrated. The excellent predictive power of the model to describe 1-fish and 2-fish trajectories was also extensively addressed in [14]. The main focus of the present work is hence not to address these questions but to study how the interactions between fish are combined in groups of more than 2 fish, while knowing very precisely these interactions for pairs of fish. Indeed, our previous work [14], while providing an extremely precise description of the interaction between 2 fish (and between a fish and the wall), offered no information about how to extend the model in bigger groups. Should one just sum all pair interactions between the focal fish and the other fish (like in physics), or only consider a very few nearest neighbor, or pick interacting neighbors at random, or instead, only consider a very few but most influential neighbors? **In the completely rewritten introduction of the Results section (see our reply to 1.11-1.12 and introductory text summarizing the main changes in the manuscript), we have made this point much clearer, including a better description of the model** (how it was built; its relevant variables – see below; the question of how to extend it to larger groups).

**#2.2.** *The computational model does contain detail about those, from my understanding both angular as well as positional/bearing information are assumed to be exchanged, and a sort of averaging process is assumed. I do agree that the full details of the model should not be revealed in the main body, however the authors should at least mention these two pieces of information (what information is exchanged and how it is integrated).*

**A:** In the description of the model in the extended introduction of the Results section (where we have also better explained why it is "data-driven"), we have now listed and described qualitatively the different information that a focal fish needs to integrate resulting in its new heading direction.

**#2.3.** *Also some high level details on how the most influential neighbors are determined should be indicated in the main body. To be honest, the full details of this are not entirely clear to me even after reading the materials and method, perhaps because this is the detailed subject of another publication. The type of information that should be present in the main body is: are influential neighbors determined at every timestep (therefore the topology is dynamic), or only at the beginning of each experiment (fixed topology)? Are those determined based on the computational model? (from my understanding the computational model is "simulated" by faking the interaction with all the other agents, determining the effect of each interaction and only choosing the k neighbors that have the largest effect, if my understand is right a description of this sort, very high level, should I believe be included in the main body).*

**A:** We have clarified the definition of the influence of a neighboring fish j on the heading change of the focal fish i, and described how the model proceeds to integrate this information. **The extended introduction of the Results section now provides a very precise definition of "influential neighbors" along with a brief paragraph about its biological interpretation: "To prevent cognitive overload, a reasonable assumption is that individual fish filter the information from their environment and thus limit their attention to a small set of their most salient neighbors [25-27] (to be followed; or to be avoided, by moving**

**away or by aligning their headings), making the notion of most influential neighbors quite natural."**

**The end of discussion now addresses the biological relevance of keeping track of a very few neighbors in more details.**

**#2.4.** *Linked to the previous point, I think the related work is missing few references that are strongly related. For example, these two papers and possibly others (dealing with the interaction topology in fish) could be referenced in the discussion at paragraph line 59 to 84:* *https://www.pnas.org/content/112/15/4690*
*https://www.ncbi.nlm.nih.gov/pubmed/21795604*
*while other models that do include physical constraints (e.g. those only based on attraction repulsion that consider non-instantaneous relaxation of the heading direction) could be mentioned as those have also been tested on robotics platforms:* *https://link.springer.com/article/10.1007/s10955-014-1114-8*
*https://journals.sagepub.com/doi/10.1177/1059712312462248*

**A:** We thank the Reviewer for his suggestions. The work by Katz et al. (2011) was already cited in the previous version of the manuscript. In this article, the authors have used force map method to visualize the effect of social interactions on fish behaviour. However, as we have recently shown (Escobedo et al. submitted), this method has a limited capacity of explanation and prediction, being rarely suitable for a direct implementation in a concise and explicit mathematical model. The paper by Rosenthal et al. investigates the propagation of behavioural changes in the context of collective evasion in a school of fish that is a quite different situation than a schooling behaviour without perturbation. Nonetheless we have included this reference in the revised version of the paper: *"In golden shiners (Notemigonus crysoleucas), another work has sought to reconstruct the visual information available to each individual (Rosenthal et al., 2015)"*

The other two articles are about swarm robotics but in a context that is very different from the one used in our paper. Both articles present different flocking algorithms implemented in groups of robots. While these works are interesting, they concern very different and very distant questions from the ones discussed in our article. Indeed our goal is not to build a flocking algorithm to coordinate the collective motion of a group of robots. Instead, we use groups of robots to measure the robustness of different local interactions strategies that can be used by fish in the presence of physical constraints, noise and speed adjustment on the coordination of collective group motion.

References cited:

Y. Katz, K. Tunstrøm, C. Ioannou, C. Huepe and I. D. Couzin (2011). Inferring the structure and dynamics of interactions in schooling fish. *Proc. Natl. Acad. Sci. USA* 108(46):18720–18725.

Rosenthal, S.B., Twomey, C.R., Hartnett, A.T., Wu, H.S. and I.D. Couzin (2015). Revealing the hidden networks of interaction in mobile animal groups allows prediction of complex behavioral contagion. *Proc. Natl. Acad. Sci. USA* 112 (15), 4690-4695

Escobedo, R., Lecheval, V., Papaspyros, V., Bonnet, F., Mondada, F., Clément, S., & Theraulaz, G. (submitted) A data-driven method for reconstructing and modelling social interactions in animal groups.

**#2.5.** *When assessing whether both models achieve good matching with the experimental data, it would be great if the authors could devise a method to quantify this numerically, for example a measure of the overlap between the two distribution (empirical vs model) for each of the metric. If this can be done it would make the description of the results much stronger and it would be possible also to support claims on what type of interaction better reproduces the data, on when there is no longer a statistically significant difference (e.g. between k=2 and k=3, etc ...).*

**A:** In the original manuscript, we only made qualitative (yet, clear and straightforward) statements when comparing probability distribution functions (PDF) of the same quantity obtained in different context (fish model/robots; interaction strategy and number k of considered neighbors) to the corresponding PDF for fish experiments. We are now supplementing our study with Table 1 (fish model) and 2 (robot platform) which report the Hellinger distance (see the last section of Materials and Methods) between a PDF obtained within the fish model/robot platform and the corresponding PDF obtained in fish experiments. As explained in the last section of Materials and Methods, the Hellinger distance has also a nice interpretation in terms of the *overlap* of two PDF. These tables certainly support our qualitative statements while putting them on a firmer ground (due to the constraints imposed for the submission manuscript, these tables are listed after the main figures, but they should appear within the text if our work is ultimately published).

**#2.6.** *I find the section collective motion in unbounded space quite important to assess the generality of the findings. Here, the paper could be made much stronger by testing the models on different conditions, most importantly on much larger school sizes, to test scalability. Although it is understandable that the data is restricted for practical reason to N=5, the computational model can be instead tested on much larger swarms. For example, I would be very curious to know if the mechanism that relies only on the most influential neighbor is sufficient to generate collective motion for large schools too. Still in the section on unbounded space, I think it is important here to report and discuss the degree of polarization, as in this setting this may be the most important metric.*

**A:** This question is indeed crucial and we have started working on the collective behavior of large model schools depending on the intensity and range of the attraction/alignment interactions and on the interaction strategy and the number of neighbors considered. This study requires extensive simulations and is beyond the scope of the present work and will be presented elsewhere in a work focusing only on the properties on the model for large schools (phase diagram of collective phases in the spirit of Calovi et al., 2014). Yet, following the referee's suggestion, we present new partial results in in the new Fig. 10 (and in Fig. 9) pointing to the cohesion of groups of size up to N=70 fish when the most influential neighbor is considered. The new Fig. S7 illustrates the failure at producing compact groups when using too small numbers of nearest neighbors compared to N/2. We have added the degree of

polarization in the analysis and the discussion as we did with the group cohesion (new figure, Fig. 10 ABDE, equivalent to Fig. 9 for cohesion).

These results are commented in a new section *"Collective motion of larger groups in an unbounded domain".*

**#2.7.** *I found the Discussion section quite weak, in the sense that it mainly re-iterates through all the results, in less details, but roughly in the same way as the Results section. I think the Discussion section should instead focus on discussing the wider implications of these results, and relating this to what is already known in the field. Having said this, I do admit that the description of the results (as done in the discussion) was indeed very useful, so this should not be discarded, but the authors should avoid discussing the results twice (between the two I would keep the result description currently done in Discussion and not the one done in Results).*

**A:** We have rewritten parts of the discussion to put our results into a more general context including the few other works that have addressed similar questions in birds and fish (see our detailed answer to the point #1.3 raised by Reviewer 1). In addition, the introduction to the Results section and the end of the final discussion now addresses the biological relevance of the notion of influential neighbors and of keeping track of only a very few neighbors. Since the detailed analysis of the collective properties of groups of model fish and robots vs actual fish requires a lot of introductory materials (now summarized in the introduction of the Results section), we have chosen to maintain the detailed analysis in the Results section. The discussion now only mentions the most striking findings of this analysis (we agreed that our previous discussion included too many details).

**#2.8.** *The school size N=5 is declared quite late in the paper, but is needed from line 127 onwards, where I kept on wondering why only k=1, 2, 3 etc were used and kept on asking myself how large was the total school.*

**A:** We have added the explicit value N = 5 at the beginning of the Results section. Note that a first mention is made in the abstract, and then in the 4th paragraph of the general introduction. Anyway, the new introduction of the results section now clearly introduces the main ingredients of our study (see reply to point 1.11) and provides a description of all the important terms and notions.

**#2.9.** *Line 225-226: this sentence is quite vague, it would be best to specify which metrics are being referred too here (all of them?)*

**A:** Changed; the text now reads: *"For the three strategies, the 5 measures on collective behavior...".* We have now avoided such vague terms in the discussion of our results, along with a precise quantification of the agreement between PDF (see Table 1 and 2 and the last section of Materials and Methods).

**#2.10.** *Line 557 onwards: please discuss more the details of the triangulation method, because I could not understand how this was done with only two measurements (normally at least 3 are required).*

**A: In our work, we did not use the infrared transmitters and receivers to measure the relative position and orientation of neighbors with a triangulation method (a camera above the setup was used, coupled to a real-time tracking software).** This is now much better explained in the revised version of our paper (description of the experimental platform); see also our detailed answer to the question #1.1 of Reviewer 1 and the new Fig. 13. We plan in a future work to use the robots' sensors to measure the neighbors' position. Obviously, using only two receivers is not enough to locate the position of a neighbor. The focal robot has to move during the measurement of its neighbors. The focal robot first measures the neighbor's infrared signals by its two receivers. Then it moves a new position not far away, which can be calculated by the its speed. Then, the focal robot measures the same neighbor again. With old and new measurements in different positions, the robot can thus calculate the position of its neighbors thanks to its microprocessor. However, all these procedures are not used in our work, a fact which is now made clearer.

**#2.11.** *Line 666: is the ref to Fig1C here right? (it is showing the fish trajectory and not the "decision process of the agent".*

**A:** Yes, it's correct. We consider that the trajectory is the sequence of points (kicks) where a fish makes its decisions, so that a trajectory is a representation of the successive decisions of the fish. We have made clearer that the fish "kicks" can be treated as decision points, an issue thoroughly addressed in [14].

**#2.12.** *What is the meaning of the three components of decoupling of the computational model? (Eqn 8 to 13). The authors should briefly outline what each of these three functions are for the two components of the model.*

**A:** These functions are the result of applying a sophisticated procedure of extraction to the experimental data of *H. rhodostomus.* This is explained in detail in Calovi *et al*., 2018. The procedure is based on the mathematical model described here, on the hypothesis that the contribution of each state variable can be decoupled from each other, and on physical principles of symmetry of the angular functions, as explained also in Escobedo *et al.* (submitted). The form of the functions is thus determined by the procedure. We have added a short text explaining that these functions stand for the attraction and alignment contributions of a neighbor to the heading variation of a focal fish, but more details should be found in the cited references. In the new introduction to the Results section, we also qualitatively describe the ingredients of the model and the relevant interactions.

**#2.13.** *Line 756 (and also in other places afterwards): how does being Object-Oriented Programming qualify the software platform developed for Cuboids ? I do not object the fact*

*that OOP was used instead of simple procedural programming, however I do not understand how this qualifies the type of architecture, and why this was chosen as a name.*

**A:** The OOP programing is a powerful program tool available in LabVIEW that we used to perform the control software for each robot in the main computer (see the new Fig. 13), because OOP can easily separate the information about each robot in one computer. We modified Fig. 13 to show the individual robot control architecture, and this architecture is implemented by OOP programming. Obviously, we have modified the text to emphasize the fact that OOP is a program tool, not a program name. To emphasize this point, we have modified the following sentence *"We use LabVIEW object-oriented programming (OOP) tool to design the distributed control software for the Cuboids robots (Fig. 13)."* In the beginning of the section "Implementation of the behavioral model in the robots". The description of our robot platform and experiments has also been extended and improved.

**#2.14.** *Putting aside the extra complications arising from having a physical platform (obstacle avoidance, finite state machine for achieving the kicking behavior etc), is the model used with Cuboids an adaptation of the same computational model studied earlier? This should be specified (even in the main text), even if very briefly.*

**A:** The Cuboids robots use exactly the same computational model as the one that is investigated with the software simulation. Only some model parameters have been changed (see Table 1) to accommodate for the different distance and time scales in robot experiments compared to real and model fish. The main difference between the model simulations and the robot experiments is that the robots need a specific *control* procedure to resolve collisions and to avoid getting stuck. The model of point-like particles and even real fish experiments (where fish swimming in shallow water can cross each other in the truly 3D experimental setup) obviously do not encounter this issue (we have added a remark concerning this point). **We have added a new brief introduction to the Results section devoted to the robot experiments which clarify all these points.** We have also modified Fig. 13 to explain that the robot experiments and the software simulation SPP use the same computational model. But the robots use the Real Time Control and Motion Control modules that work in parallel with the computational model. The description of the swarm robotics platform has been also extended and improved.

**#2.15.** *Information contained in the section starting at 823 comes somehow too late and contains some overlapping information with what was said before, with some important details added. I think this section should disappear and the content be merged and those important details added earlier where relevant. Or the section could be moved earlier and include also the information presented somewhere else (for example how to calculate the most influential neighbors).*

**A:** We thank the Reviewer for his suggestion that definitively improves the readability of the manuscript. We have removed the section and merged the information as recommended by the Reviewer. The characterization of the three strategies appears now much more clearly at the beginning of the thoroughly rewritten introduction to the Results section (see our reply to 1.11-1.12 and the introductory text summarizing our changes).

## Reviewer #3:

*The manuscript "Computational and robotic modeling reveal parsimonious combinations of interactions between individuals in schooling fish" implements rules of interaction previously deciphered between pairs of fish into a model simulation and robotic swarm with the aim of determining the structure of the interactions in real fish. The paper seems to be the first attempt at taking rules directly inferred from real animals and implementing them into robot systems. This makes the paper particularly novel and interesting and will make an important contribution to self-organization, bio-inspiration and collective behavior. However, there are a number of important issues that I would like to raise before the paper can be accepted for publication.*

*General comments:*

**#3.1.** *While I was very impressed by the interdisciplinary approach to the topic, I was a little disappointed with the lack of statistical comparison between the real data and the simulation and robotic results. Many statements are made without any comparative approaches, and in some cases, my interpretation of the results appears different from the authors. It would relatively simple to implement tests such as Kolmogorov-Smirnov tests, that can statistically compare the distributions of the variables (PDFs) that the authors have computed between the fish, model, and robots. This would allow the authors to make more quantitative comparisons and to more accurately identify which strategies capture the real fish's behaviour.*

**A:** We thank the Reviewer for explicitly sharing his/her interest on our interdisciplinary approach of collective motion in fish schools. We agree with the referee that adding a quantitative comparison between the real data and the simulation and robotic results would improve the readability and the overall strength of our paper. The Reviewer #2 has also recommended us to include a quantitative comparison between theoretical and experimental results. We refer the Reviewer #3 to our reply to the point #2.5 by Reviewer #2. The results concerning the Hellinger distance between fish experiments PDF and the corresponding PDF produced by the model and the robots are given in Table 1 and 2. The notion of Hellinger distance is detailed in the last section of Materials and Methods.

**#3.3.** *It would be particularly useful to note the best-supported model (i.e. nearest, random, influential, for k = 1, 2,3 etc.) for each measure that the authors calculate. For example, the authors could identify in a table the (statistically) best-supported model for group cohesion, group polarization, mean distance of individuals to the border, etc. This would allow readers to identify consistently (across different metrics) chosen models that are supported by the data. As the authors suggest, this could be models that only use one or two influential neighbours, but this needs to be tested. As the manuscript currently stands, readers cannot integrate all the information that the authors have produced in order to determine which models are superior over others.*

**A:** We agree with the Reviewer. As mentioned in our answer to the previous point we have introduced a distance between PDF to measure the similarities between the real fish experiments and the simulations of the computational model on one hand and the robotic experiments on the other hand for the different quantities that we used to quantify collective motion. The results are provided in Table 1 and Table 2. We also refer the referee to the answers we provided to the question #2.5 that was formulated by the Reviewer #2.

**#3.4.** *I also found the paper particularly difficult to follow in places when figures, and importantly the subpanels within the figures, were not referred to in the results section. For example, lines 208-211 discuss polarization without a reference to the polarization figure. This is not the only example. There were also many relevant and important lines (curves) missing from the figures, which made interpretation impossible (see my specific comments below).*

**A:** We thank the Reviewer for pointing out these mistakes in the figures, which have now been corrected. We have also added the references to the corresponding figures, panels, and tables in the discussion of the results. Overall, the Results section has been hopefully greatly improved (style, clarity, notations…), in particular thanks to a new introduction to the Results section and a new brief introduction to the section devoted to the results of the robot experiments.

**#3.5.** *The authors argue in the discussion that responding to a small number of neighbours could represent selection attention mechanisms, thereby reducing the amount of information that an animal needs to process. However, from my understanding, the implementation of the 'most influential' neighbour rule appears to require a focal individual to calculate the influence that all neighbours have on that individual, and then choose the neighbour with the most influence. This does not, therefore, reduce the information an individual needs to process. I would like to see the authors address this point in the manuscript.*

**A:** We thank the Reviewer for pointing out this issue that is now discussed in detailed in the revised version of the manuscript. The model accurately calculates the impact on the behavior of a focal fish of the relative position, distance and relative orientation of each of the neighboring fish that are in the vicinity of the focal fish. On this basis, it is then possible to determine which of its neighbors exert the highest influence on a focal fish, that is to say to rank the influence of the neighbors. The referee is right to point out that in order to determine which of its neighbors exerts the greatest influence on a focal fish, one first needs to acquire information on the neighbors and then calculate the influence exerted by each of them on the focal fish. In vertebrates, and in particular in fish, these *filtering* operations are routinely done by the midbrain and forebrain networks that are carrying out computation in parallel to process the visual information and select the most salient stimuli that are the focus of attention [Knudsen, 2018]. In particular the midbrain network and especially the Optic tectum continuously monitors the environment for behaviorally relevant stimuli. This is a primary site where the information about the neighbors is filtered for cognitive decision. Then the forebrain network selects those stimuli on which the fish focus its attention. The local interaction strategies that we have investigated in our paper correspond to the different ways for an individual to focus its attention on the stimuli.

Then when we argue in the discussion that *« responding to a small number of neighbors could represent selection attention mechanisms, thereby reducing the amount of information that an animal needs to process »* we are referring explicitly to the attention paid simultaneously by a fish to different neighbors after the visual information by the midbrain networks to identify the most salient stimuli has been processed (i.e.: ranking the influence exerted by the neighbors on a focal fish). We have introduced the following paragraph in the discussion to make this point clearer (line 503 of the previous version): *"In vertebrates, and in particular in fish, the midbrain and forebrain networks are carrying out computation in parallel to process the visual information and select the most salient stimuli that are the focus of attention. The midbrain network continuously monitors the environment for behaviorally relevant stimuli (Knudsen, 2018). This is a primary site where the information about the neighbors is filtered for cognitive decision. Then the forebrain network selects those stimuli on which the fish focus its attention. The interaction strategies that we have investigated in our paper correspond to the different ways for an individual to focus its attention on the stimuli (i.e. the neighbors). Our results show that each fish interacts with one or two neighbors that are the most salient and this reduces the amount of information that needs attention. So each individual must acquire a minimal amount of information about the behavior of its neighbors for coordination to emerge at the group, thus allowing individuals to adapt to information overload when they move in large groups [31]."*

Reference cited: Knudsen, E.I. (2018) Neural Circuits That Mediate Selective Attention: A Comparative Perspective. *Trends in Neurosciences*, 41: 789-805.

We also added a paragraph in the introduction of the Results section, around the introduction of the 3 strategies:
*"To prevent cognitive overload, a reasonable assumption is that individual fish filter the information from their environment and thus limit their attention to a small set of their most salient neighbors [25,27] (to be followed; or to be avoided, by moving away or by aligning their headings), making the notion of most influential neighbors quite natural.*
*…*
*These interaction strategies explore different ways for an individual to focus its attention on the most relevant stimuli (i.e., neighbors)."*

**#3.6.** *In relation to the most influential neighbour, it appears from Movie S7 that for large portions of the time (perhaps the majority of the time) most individuals are following a single neighbour - the neighbour that is closest to the wall and perhaps making the largest contributions towards angular differences between the robots. Is this also observed in the model simulations? Could the authors discuss what implications this could have on distributed decision-making, if particular individuals are disproportionally influencing collective movement?*

**A:** During the first 8 seconds of the video, because of particular configuration of the robots within the arena, robot number 26 adopts a role of temporary leader. However, at t=9 s, robot number 26 is then replaced in this role by Robot 31. Such a turnover is also observed in simulations. In fact, in our system, no particular individual (fish or robot) is playing the role of a leader that could have a disproportionate influence on the others. We plan to explore in the future the implications of individual biases in the sensitivity of social influence but this is

well beyond the scope of our paper. Note that a notion of "leadership" was studied in [14], finding a good agreement between our model and the dynamics of pairs of real fish (properties of the "leader" vs that of the "follower"). It was in particular shown that, within the model, and despite all agents being equivalent, an apparent leader can persist for long times in the dynamics of pair in agents.

**#3.7.** *In general, I would also like the authors to be careful with some terminology. The authors refer to groups being 'better' or 'worse' in terms of cohesion or polarization. Please simply state the direction of effects.*

**A:** Amended. We now avoid any such vague statement in the discussion of our results. In addition, our analysis has been made much more precise by the introduction of an objective quantifier of the similarity between two PDF (see the new last section of Materials and Methods, and Table 1 and 2; see also our answer to #2.5).

Other comments:

**#3.8.** *Line 109: Remove "a".*

**A:** Removed. Now the text reads: *"...neighbors for coordination to emerge..."*

**#3.9.** *Line 112: Change "basically" with (i.e. one or two).*

**A:** Changed**.**

**#3.10.** *Line 147: remove "huge"*

**A:** Removed.

**#3.11.** *Line 228:230: This statement does not seem to be supported by the data. Counter-milling is observed when individuals interact with a nearest neighbour only. Figure 9 shows evidences of this, as does Fig. S5. Please rephrase.*

**A:** The Reviewer is absolutely correct. In fact, the 3 strategies lead to comparable PDF for the counter-milling index Q for k=1 interacting neighbor (Fig. 8; previously, Fig. 9), as quantitatively confirmed by the results of the new Table 2. In particular, for the point raised here by the Reviewer, the corresponding sentence now reads "*Counter-milling is obtained for the three strategies with comparable PDF (Fig. 8; see also S5 Fig.), quite similar to the one obtained in fish experiments (we will see that the agreement unfortunately worsens when increasing k; see Table 2)".* We have also given a possible interpretation for the fact that counter-milling is stronger in the model and robots for k=2 and 3 compared to fish experiments and k=1. Note that the general discussion of our results has been much improved (style, clarity, precision, notations…).

**#3.12.** *Line 235: The line of k = 1 for the most influential neighbour in Figure 6C appears to be missing, making this comparison impossible.*

**A:** The Reviewer is right; we have corrected this figure. We thank the reviewer for pointing out this important mistake.

**#3.13.** *Line 236: I do not see any evidence that if a fish interacts with a random nearest neighbour, that group cohesion is 'better' than if they interacted with a nearest neighbour. There does not appear to be any differences in the shape of the curves in Fig. 4 A and B. Further, Fig. S5 appears to be a referring to the wrong figure here. Again, statistical comparison would aid in data interpretation here.*

**A:** The curves corresponding to k = 1 in Figs. 4A and 4B show that very weakly cohesive groups (with C > 15 cm) are much more frequent when fish interact with the nearest neighbor than with a randomly chosen one. Table 2 confirms that, for k=1, the nearest neighbor strategy leads to the worst agreement with fish experiments for the cohesion, while the most influential neighbor strategy convincingly results in the best overall agreement (especially for the cohesion C and the polarization P). This is even more true for robots where the nearest neighbor strategy completely fails at producing cohesive and polarized groups (see our interpretation in the improved discussion of the results).

As for the figure label, the reviewer is right and this mistake has been corrected.

**#3.14.** *Line 248: Again, this statement does not appear to be supported by the data. Groups are more cohesive and more polarised when k = 2, than when k = 1 across all strategies.*

**A:** The discussion of the results (in particular, for k=2) has been thoroughly revised and the precision of the analysis (as well as the general style and presentation) has been drastically improved, in particular by exploiting the results of Table 1 and 2 for the Hellinger distance between model/robot PDF and the corresponding PDF for fish experiments (see also the new last section of Materials and Methods).

**#3.15.** *Line 250: Coincide more frequently with what?*

**A:** Again, we have revised the discussion of our results to systematically avoid vague statements, and by referring to the results of Table 1 and 2 to justify our qualitative statements.

**#3.16.** *Line Again, the line appears to be missing for k = 3 appears to be missing from Fig. 9c.*

**A:** The reviewer is right; we have corrected this figure. We thank the reviewer for pointing out again this mistake.

**#3.17.** Line 364: There doesn't appear to be any groups that are larger than 18 cm in Fig. 4D? Similarly there are no groups that are further than 22 cm from the border in Fig. 6D.

**A:** The spatial values are scaled by the factor lambda_R = 0.35 (in robots) for comparison purpose, so that the values of distance for robots in the Fig. 4 and 6 should by divided by 0.35 to recover the actual distances. To avoid any confusion, the new version of the manuscript only mentions distances as expressed after this rescaling (see our discussion of the rescaling in our answer to point #1.18; see also the paragraph addressing this point in the introduction to the results for our robot experiments).

**#3.18.** *Figure 10A: Again, the line for k = 0 appears to be missing from the plot. This may be because cohesion reduces rapidly, but then this should referred to in the caption. The scale on the x axis for subplots D & E should be the same.*

**A:** We have modified the text of the caption. Actually, the line is not missing but is so vertical that it overlaps with the vertical axis and cannot be seen on the scale of panels AB (however, see panel C).

Regarding the x-scale of panels DE, the scale of panel D permits to clearly see the slight difference between the different curves for k=1, to 4 (all with a $d_{cut}$ of order 0.8m). For the nearest neighbor strategy (panel E), the scale is adapted to the fact that, for k=2, $d_{cut}$ =3.5 m is 4 times larger than for the most influential strategy. We do not think that the different scales would confuse the reader, and consider it is best to adapt the x-axis scale to the relevant range of $d_{cut}$ for the 2 strategies.

**#3.19.** *Line 530: Were the experimental fish used more than once? Please add details.*

**A:** We added the following information: *"We performed 11 trials with groups of N = 5 fish, and for each trial, we used different fish taken from the breeding tank"*

**#3.20.** *Line 527: 60 mm not 6 m !*

**A:** Yes! Corrected.

**#3.21.** *Line 558: 'Peering angle' is undefined, and doesn't appear to be depicted in Fig. 2. Please clarify.*

**A:** The viewing (we dropped the term "peering") angle is psi_ij, the angle with which the focal fish i perceives its neighbor j (*i.e.*, The angle between the velocity of i and the vector between i and j). This definition (as well as that for other relevant variables that a fish integrates in its decisions) has been made much clearer in the revised introduction to the Results section and in Fig. 2.

**#3.22.** *Line 564-565: This sentence is unclear. How many time-steps is required before a neighbour's id is deleted from the list?*

**A:** We agree with the Reviewer that this sentence is not clear and can be confusing for the reader. We removed the two following sentences: *"Moreover, each robot keeps a list of its neighbors with this information that is updated with time. If the robot does not receive the IR signal transmitted by a neighbor who is already in the list for a long time, the item related to this neighbor is deleted from the list".* We have also better explained how the robots were used in the experiments. Even if the robots can move in an autonomous mode, we did not use the infrared transmitter and receiver to measure the relative position and orientation of neighbors by triangulation method. This is because the robotic experiments were used in a remote control mode to investigate the impact of collision avoidance and the physical constraints on collective movements (see new Fig. 13). We also refer the referee to the answers we provided to the question #1.3 and #1.4 formulated by the Reviewer #1.

**#3.23.** *Robots and Experimental Platform sections: It is not clear to me whether the robots are using the infra-red signals from neighbouring robots to inform their decisions to move. Given that a whole section (lines 553 – 572) is dedicated to explaining robot-robot sensing, I would suspect so, but it is not clear given that the computer software is providing the robots with control signals. These sections, along with Figures 12 and 13 need significant clarification. To start, it may help moving paragraph beginning line 595 to the start of the*

*experimental platform section. It may also help explicitly stating that infra-red communication is not-used (if it isn't) to inform individual robots movements when describing this earlier on.*

**A:** As we mentioned previously, we did not use the infrared transmitters and receivers to measure the relative position and orientation of neighbors by triangulation method. This section was only intended to provide the reader with a complete description of the robotic hardware. We plan to control our robot autonomously in future works. We have modified the description of the robotic platform in the Method and Material section, to prevent any confusion and have introduced a new Fig. 13. We also refer the referee to the answers we provided to the question #1.3 formulated by the Reviewer #1.

**#3.24.** *Figures 12 and 13 would benefit from consistent terminology and highlighting what the coloured regions represent. i.e. What does the orange section of this figure represent? What is the red section?*

**A:** We thank the Reviewer for his suggestion. We found that using different colors to represents different functions was a good idea. We thus modified Fig. 12 and 13 and used the same colors to represent the same functions in these figures to help the reader to understand the program that controls the robot. In these figures, the yellow color indicates the computer, the orange color indicates the image processing, the measurement of the environment and the neighbors, and also the computational model, and the red and pink colors indicate the hardware in loop simulation including the real-time control and motion control modules.

**#3.25.** *Figure 13: "for each robot based [on] its local information"*

**A:** Changed.

**#3.26.** *Line 629: mins not (mn).*

**A:** Changed.

**#3.27** *Line 631: Remove this sentence as the kick are not independent and therefore the this is not 16 hrs of independent data, which it could be misconstrued as.*

**A:** Changed.

**#3.28** *Line 634: Surely the local minima (not maximum) is defined as the onset of a kick?*

**A:** Changed.

**#3.29.** *Line 637: The authors 'double their data' by creating the 'mirror-image' of the trajectories. This represents a form of pseudo-replication and should be avoided. Why can't the authors simply compare the absolute distributions (e.g. absolute rotation index between the real data and the simulations/robotic results)?*

**A:** Our justification for the symmetrization procedure was indeed misleading. It now reads: *"In {Calovi2018}, no statistically meaningful left/right asymmetry in the trajectories of single fish (300000 kicks recorded) or pairs of fish (300000 kicks recorded) was observed. Hence, for any observed trajectory, the mirror trajectory (that is the same one, but as observed from the*

*bottom of the tank instead of from the top) would have exactly the same probability to be observed. Assuming the absence of left/right asymmetry for groups of 5 fish (as observed for 1 and 2 fish), leads to the same conclusion. Groups of 5 fish (as well as groups of 5 model fish or 5 robots) rotate clockwise (CW) or counter-clockwise (CCW) around the center of the tank for long periods (collective U-turns in groups of 2-20 fish have been studied in {Lecheval2018}). Therefore, for the much shorter present fish (and especially robots) experiments compared to {Calovi2018} (60312 recorded kicks, instead of 500000), one would observe an artificial asymmetry (groups turning more often clockwise than anti-clockwise or the opposite) only due to the lack of statistical sampling of the rare collective direction changes. In order to avoid this artificial asymmetry, for each set of 5 trajectories (fish and robots), we have added the mirror set (the trajectories as seen from the bottom of the tank). Again, this procedure is perfectly sound once the absence of left/right asymmetry observed in very long 1- and 2-fish experiments is reasonably assumed to hold in our present 5-fish experiments (the model and its version implemented in robots have obviously no left/right asymmetry, per construction).* ***Note that only the distribution of theta_{w}^B is affected by this symmetrization procedure,*** *and not the distributions of group cohesion, polarization, distance to the wall, counter-milling index (the latter a relative quantity), which are invariant by the mirror symmetry."*

**#3.30** *Line 660: "Decisions of the fish in the simulations and the decisions of the robots"*

**A:** Changed.

**#3.31** *Figure 2: line r_wi is not horizontal. Psi_ij is not represented in the figure (Psi_ji is, not this is not referred to in the caption).*

**A:** r_wi is the distance from the individual i to the border of the arena. The line between a fish and the nearest wall has no reason to be horizontal. We have added the word "nearest" to the caption.

The reviewer is right: what is represented is psi_ji and not psi_ij. The reason for this is that the angle psi_ij would be close with the angle theta_wi in the configuration depicted, making the figure difficult to read. We have chosen to show the angle psi_ji, and now have clarified that both angles psi_ji and psi_ij are not necessarily equal.
