# Supplementary material for: Computational and robotic modeling reveal parsimonious combinations of interactions between individuals in schooling fish
Source: PLoS Comput Biol. 2020 Mar 16;16(3):e1007194. doi: 10.1371/journal.pcbi.1007194 (PMC7098660; doi:10.1371/journal.pcbi.1007194)
Supplement: Attachment — Submitted filename: Rebuttal_Letter.pdf [file pcbi.1007194.s025.pdf]

The reviews agree on the substantial improvements on the text, but few amendments are still necessary. In particular, pay attention to define properly the contribution of the paper, and to the motivations and contributions provided by the usage of a centralised multi-robot system.

We wish to thank the editor for considering our manuscript and the referees for their constructive comments, which have helped us to further shape this manuscript for publication in *PLoS Computational Biology*. We hope that this second revision also meets the expectations of those reviewers that were not fully satisfied by the first revision.

Reviewer's Responses to Questions

Comments to the Authors:

Reviewer #1:

The authors have carefully prepared an extensive revision of their paper. The quality has certainly increased and many of my previous concerns have been resolved. I still see a few issues that would need to be resolved.

#1.1 *"most influential strategy" with k=1 neighbors: Thanks to the improved paper I have a better understanding of the "most influential" strategy now. What I had flagged as "potentially self-referential" before is not yet resolved but I can clarify my concerns now. What's still unclear from the text, I think, are the temporal aspects of the strategy and what information needs to be available at what time. The mathematical description (eqs. 4 and 9) could be improved accordingly.*

Answer: We thank the Reviewer for asking for a clarification about this important point. We agree that it was not clearly explained in the text and have added a more detailed description of what a "time step" is, from the beginning of Section "Computational model" to Eq. (4). We have introduced the "kicking time" variable $\boldsymbol{t_i^n}$ to denote the onset of the n-th kick of agent i. We have also made clear that an agent uses the **instantaneous** positions and headings of the other agents at the onset of a new kick to decide of its new headings and that the motion of the different agents is **asynchronous**, meaning that the kicks for different agents have no special reason to occur at exactly the same time.

Thanks to the new formulation (and other changes shown below), Equations (4) and (9) are now easily understandable and are left unchanged.

We have also expanded the main text (right before Sec. "Collective behavior in fish experiments", L. 212-226, specifying that

> *"The strategy is thus characterized by the number k of neighbors taken into account in the social interaction and the criterion used to select them (NEAREST, RANDOM, or MOST INFLUENTIAL). The strategy remains unchanged along the whole simulation. However, the identity of the neighbors selected to interact with a given agent can change from one kick to another, and must be updated at each kicking time of this agent. For instance, when using the NEAREST strategy with k = 2 in a group of N = 5*

agents, the agents taken into account in the social interaction in the n-th kick of agent 1 can be the agents 2 and 3, and the agents 4 and 3 in its (n + 1)-th kick. In order to select these k neighbors at a specific kick, the N−1 agents must be sorted according to the criterion corresponding to the strategy used in the simulation. This sorting process is carried out at each kicking time of the focal agent, independently of the state (kicking or gliding) of the other agents. If N is so large that the computational cost of this process becomes prohibitive, a more efficient algorithm can be implemented, such as keeping track of the agents that were selected in the most recent kicks and exploiting grid algorithms to identify neighbors"

Also note that a given strategy is kept fixed during each simulation. This means that the **number** and the **kind** of neighbors (depending on the strategy considered) taken into account in the social interaction remain unchanged along the simulation, but the **identity** of the neighbors (i.e., agent 2, agent 4, agent 5...) can change from one kick to the next. The information needed is the relative position and heading of all the N–1 neighbors of the focal agent, which must be updated at each kicking time of the focal agent.

This is now explained four lines after Eq. (4), where the notation <j,i> used in the sum of social interactions in Eq. (4) is now more clearly described (lines 1027-1035):

"The notation <j,i> indicates that the sum is performed over all the agents j considered to interact with agent i. The number k of agents considered to interact with an agent is part of what constitutes a social interaction strategy, and remains constant along the whole simulation. When $k < N-1$, the identity of these agents depends on the strategy, but also on the instantaneous state of the system, so that their identity must be updated at each kicking time of the focal agent i. At each kicking time t_i^n, the agents are sorted according to the criterion used in the strategy of interaction: the distance to the focal agent $d_{ij}(t_i^n)$, a random selection of neighbors, or the influence on the focal agent $I_{ij}(t_i^n)$. Once sorted, the k first agents are considered in the sum in Eq. (4)."

See also the answers to the sub-points 1.1.a-1.1.c below.

#1.1.a *most influential over what time period?*

It would be possible that a fish picks a neighbor that is most influential over its lifetime, experiment duration, or a single time step (whatever that would biologically mean), etc. Most influential during lifetime and experiment could only be determined posteriori, hence is probably not useful. I believe you mean most influential in the current time step. This is only implicitly stated in the paper (or I missed it). Please clarify. I'd recommend to extend the mathematical description, such that an explicit maximization operation over all neighbors for a current time step is given. Something like: for given time step t most influential neighbor j of fish i: j(t) = arg max_j I_{ij}(t)

A: The neighbors taken into account to calculate the new heading direction are updated at each new kicking time (the onset of a new kick). This means that the interacting neighbors can change from one kick of the focal fish, to the next kick of this same fish.

We have clarified this point, by explaining that the neighbors taken into account to evaluate the social interaction are calculated at each kicking time by means of a procedure that sorts the neighbors according to the criterion used in each strategy (the distance to the focal agent, a random sorting, or the influence exerted on the focal agent).

See the new text in the Section "Computational model" (L. 975).

#1.1.b *most influential neighbor selection is valid for how long?*

It would be possible that a fish picks a most influential neighbor for a duration of its lifetime, experiment duration, or a single/multiple time steps. I believe it's only valid for 1 time step. However, that means that each fish i has to check each potential neighbor j in each time step t whether it is its potentially most influential neighbor; which leads to issue 1c.

A: We think that our answer to the previous point solves this question. The Reviewer is right, neighbor selection is "only valid for 1 time step", understanding "time step" as the duration of the gliding phase of the focal agent, i.e., from $\boldsymbol{t_i^n}$ to $\boldsymbol{t_i^{n+1}}$.

#1.1.c *is the concept of k=1 misleading?*

Often in the paper you seem to argue that the most influential neighbor strategy is efficient/filtering (?) in a sense that only one neighbor is considered (e.g., line 109 "interacts only with a single neighbor"). However, each fish needs to consider all other fish in each time step to find its currently most influential neighbor. In terms of computational complexity, what does not scale is the mere consideration of all neighbors/swarm members independent of the actual calculation as given by eq. 4. Please clarify. Please also discuss that this strategy would not scale. Possibly, it makes sense to think of a two-step selection process: first selection by distance and then selection by most influential? Scaling is certainly not relevant for the investigated swarm

A: The Reviewer is right in that the calculation of the direction of the next kick of agent *i* requires to consider all the other N–1 agents. This means that, at each kicking time, the distance/index/influence of N–1 agents have to be evaluated according to the strategy nearest/random/most influential, respectively. We would like to point out that the term "considered" is used in the text in the sense of "considered in the sum term of Eq. (4)".

As for the computational complexity of the interacting neighbor selection algorithm, one has to clearly distinguish the practical problem of the implementation of the model in a numerical simulation and its potential biological relevance, and we discuss both aspects below (and in the manuscript: at the end of the introduction to the Results section, in the Computational model section, and in the Discussion).

The process of evaluating the distance and/or the influence of N agents is nowadays computationally almost instantaneous for values of N below 1000, both in the numerical simulations and in the robotic platform. The Reviewer is right in that, for even larger values of N, a selection process would be necessary to reduce the number of agents to consider, based e.g. on a two-step process (first nearest ones, then sort by influence), as suggested by

the Reviewer, or by tracking which agents were the most influential ones (or the nearest ones) in previous kicking times (an agent that is the 100th most influential at one kicking instant will hardly become the most influential one at the next kicking time). Note that there are many efficient algorithms to search for close neighbors (for instance, simple grid-based algorithms). This would be relatively easy to implement and would result again in an almost instantaneous algorithm. Note that in physics, and even for long-range interactions like gravity, the most efficient algorithms are currently able to handle up to *20 billion* particles. It is clear that simulating a "big" fish school should not be seen as a challenging computational problem, even on a single high-end PC or a small computer cluster.

From a biological point of view, as we mentioned in the final discussion (starting line 649), and briefly in the beginning of the Results section, the midbrain and forebrain networks are carrying out computation in parallel to process the visual information and filter the most salient stimuli that are the focus of attention and in particular the most influential neighbors. Moreover, one has to consider that this information filtering process may also be combined with a visual screening phenomenon, especially when fish swim within a compact school. Therefore, the strategy which consists in using only information on the most influential neighbor is particularly well suited to group swimming, because it makes it possible to minimize the information required for the fish to coordinate their swimming while maintaining cohesive groups. On the other hand, it is a perfect scalable mechanism when group size increases, precisely because of the information filtering processes that are routinely performed by the visual perception system of fish. Again, it is important to distinguish the computational complexity of a model used to account for the perceptual and selective attention processes and that of the neuro-physiological mechanisms that are involved in the acquisition, filtering and selection of information in the brain of fish.

#1.2 *central control and remote controlled robots:*

Now it is clearer that the robots are non-autonomous and remote controlled. Could you please state that very clearly in the paper? You correctly mention that Fig. 13 reflects that but Fig. 12 may even be better for that. Maybe you can indicate there more clearly that the communication by Wifi is one-way (if it actually really is) from the computer to the robots. Hence, no sensors of the robots used and the robots compute nothing onboard.

A: We thank the Reviewer for this suggestion. The WIFI broadcasting is one-way communication for sending the command of robot's action every 20 ms. Then all robots execute their commands respectively at the same time to perform the collective motion in physical world. There is no information acquired by sensors of the robot that is sent back to the computer via the WIFI channel. We emphasize this in the caption of Figure 12.

We have modified the caption of Figure 12 as follows:

> ***Fig 12. Structure of Cuboids platform****. Two main parts: the physical hardware and the control software. The hardware consists of a square platform. A camera mounted on the top of it to monitor the movements of Cuboids robots, which are controlled in a distributed way by a wireless router. The software processes the image acquired by the camera, then computed the command of actions to be performed by each robot, and finally sends the control signals to the robots via the* ***WIFI channel. Then, all the***

robots execute their commands at the same time to perform the collective motion. The WIFI broadcasting is one-way communication for sending the command to the robots every 20m. In this setup, no information acquired by robots sensors is sent back to the computer though the WIFI channel. Credits to David Villa ScienceImage/CBI/CNRS, Toulouse, 2018.

#1.3 *embodiment:*

You have added arguments of why an embodied model in the form of swarm robots is helpful. I'm certainly on your side here but the mentioned issues of collision avoidance and time-consuming turns on the spot due to non-holonomic drives could actually be simulated. Maybe it is also about imperfections in the robots and influences of the real world? Noisy sensing seems mostly excluded due to the central sensing approach.

A: Most existing research on collective motion merely tested models with numerical simulations, which ignores some physical aspects. So, if one hypothesis about the way agents combine interactions with other agents is just tested with a pure numerical simulation rather than with physical experiment, one may expect to get only some partial validation because the real-world constraints are not integrated. Moreover, it is quite hard to simulate all physical aspects with a multi-agent system. For instance, this would require an NVIDIA open source physics simulation engine like PhysX to simulate the collision of the square body of the robots. We would also need to build a virtual image processing system for the measurement of the agents' position to evaluate the impact camera noise on the model. So, instead of building a rather complex model of a virtual physical world to test our model and the different hypotheses about the interaction strategies, we chose the alternative option that consists in using robotic embodiment.

We added the following sentences after line 731, behind the last paragraph of Swarm robotic platform – Robots, in Materials and Methods, to emphasize this point:

> *"We tested the model with the robotic platform because there are many physical aspects that have to be considered to assess the robustness of the coordination mechanisms when they are implemented in a physical hardware. These physical aspects include the friction of wheels, the noise of gear box, the blurring of the camera, the wrong identification of the tracker, the delay of the communication, the overload of computation, the blocking of the onboard communication bus, the square shape collision of the robot frame, the mismatch parameters of the interaction model, the impact of the obstacle avoidance protocol, and the non-holonomic constraint of the robot. All these physical aspects have large effects on collective behavior, (especially when robots move in a crowded space) and are difficult to include in a model."*

#1.4 *real time:*

You are referring to "real time" several times. It is a bit unclear whether this is meant as "real-time control" such that you can guarantee that (each robot?) reacts within strict max. time intervals.

A: We thank the Reviewer for pointing this out. There are two levels control for the robots. The first one is a Model control which is used to determine the position of the target; in that case the control frequency is low (about 1.3 second). Another level is a Real-time control at higher frequency with respect to the real time motion of the robot, which is used to control the navigation of the robot toward the target and the obstacle avoidance (see the table of Algorithm of the Real Time Control module). The maximum time interval of real time control is 20ms for each robot. With such a fast control frequency, the communication channel is always busy. Hence, this requires that computational model, the object-oriented software and the robot hardware work harmoniously together. To solve this problem, we designed and used a specific protocol to broadcast the Motion Control command $(V_{R,i},V_{L,i})$ to each robot, respectively, based on TCP protocol, which can guarantee the speed and the robustness of communication channel.

We added the following sentences at Lines 822-833, behind the table of Algorithm of the Real Time Control module:

> *"There are two time-scale in the control for the robots. The long time scale is determined by the time taken in simulating the computational model, which is about 1.3 second. The short time-scale corresponds to the Real Time Control module, which operates at a high frequency with respect to the real time motion of the robot. This module is used to control the navigation of the robot toward the target and the obstacle avoidance (see the table of Algorithm of the Real Time Control module). The maximum time interval of the Real Time Control module is 20 ms for each robot. With such a fast frequency, the communication channel is always busy. To solve this problem, we designed and used a specific protocol to broadcast in one loop the Motion Control command $(V_{R,i},V_{L,i})$ to each respective robot i, based on TCP protocol, thus guaranteeing the speed and the robustness of communication channel. The average duration of one of these loops (for all robots) in the Real Time Control is about 13ms (see Fig. 13)."*

Reviewer #2:

The authors have now produced a much improved version of the manuscript, addressing in a careful and satisfactory way all the comments of the three referees. From my side the article is very close to publication quality. Only few comments left:

#2.1. *My very first remark about how the contribution is claimed was partially address but still unresolved. At line 97: "To better understand how individuals combine and integrate interactions ... " to me, the words "combine and integrate" recall this idea: Given we know how many neighbors the focal individual should consider, and what information is exchanged, what to do this information: summing? calculating averaging, taking the max, min? etc ... I*

do agree with what is written at line 174-178 (picking how many neighbors and how to pick are important). However, the words "combine and integrate" makes me think of what you do "after" the other two problems (picking the neighbors) are solved. In this specific situation, only one neighbor ends up being picked with a particular strategy, therefore there is not much left to "combine and integrate". I therefore only propose to make the statement more precise and aligned with what was actually done (it is possible that addressing the actual combination was either done in [14] or object of future work, I do not see how this can be simply assumed as averaging).

A: First, we have changed in the text “combine and integrate” (the information) into “integrate and combine” (lines 29, 33, 99) since the environment (other fish; obstacles) has first to be perceived and “analyzed” (i.e., integrated) by a focal fish before the effects of its different components (neighboring fish; obstacles) are combined, resulting in the next action of the focal fish.

In [14], it was found that combining the interaction of the wall and that of the other fish by simply *summing* them ultimately **leaded to a faithful model in good agreement with experiments**. Obviously, there is an infinity of other possible nonlinear combinations of both contributions but it is not clear how to build a relevant (and “natural”) one or if it would do any better than the already satisfactory and natural *additive assumption*. In the present work, when considering several neighbors selected by one of the 3 strategies, we again assume that their contributions add up (like in physics). This is clearly a natural choice, and again, it is not clear at this stage how to build a (more?) relevant nonlinear combination of the contributions of the different neighbors and of the tank wall.

Once we have made the additive assumption, our present work really focuses on the actual neighbors which are selected (the 3 strategies), emphasizing the effectiveness and possible biological relevance of the notion of MOST INFLUENTIAL neighbors allowing for compact and polarized groups even for k=1. After the selection of the interacting neighbors, their contributions are combined additively (along with the effect of the wall) to produce the new heading angle of the focal agent/robot.

Finally, it is true that the word "combine" may appear inappropriate when k=1 when only one interacting neighbor is considered at the instant of the kick (however, the generic case was clearly considered in the introduction from which the Referee extracted the sentence). Still, this contribution of the neighbor to the heading angle of the focal fish must be "combined" (meaning “added” here) with the effect of the tank wall, even for k=1, like in [14].

#2.2. *Given the new details about the robotic experiments, I do forbid the use of the word "swarm" across the whole manuscript :-) For a group of robot to be considered a "swarm", embodied sensing is extremely important.*

A: Changed. We have removed the word "swarm" from "swarm robotic platform" in the abstract, and replaced it by "group" in abstract and keywords, end of the Introduction, in Sections "Collective behavior in robotics experiments", "Swarm robotic platform" (now "Robotic platform"), and in titles and captions of figures and videos.

#2.3. *I propose to replace Fig 14 with an actual finite state machine diagram, rather than a flow chart, because this would be more aligned with what explained in the main text (it*

would represent mainly the MOVE state and COMPUTE state, etc, the other being transitions across states).

A: We thank the Reviewer for his advice. However we still want to keep the original Figure 14 in the main text because it clearly shows the two main states (COMPUTE and MOVE) of the robot. Following the suggestion we also added the supplementary S8 Fig with the finite state machine diagram. The main text now refers to "S8 Fig." together with "Fig. 14" in the Section "Implementation of the behavioral model in the robots", as in line 852:

> *"The state machine control structure for an individual robot includes two main states: COMPUTE state and MOVE state; see the flow chart of the robot state machine and the finite state machine diagram in Fig. 14 and S8 Fig respectively. "*

The finite state machine diagram is now shown in S8 Fig with the following caption:

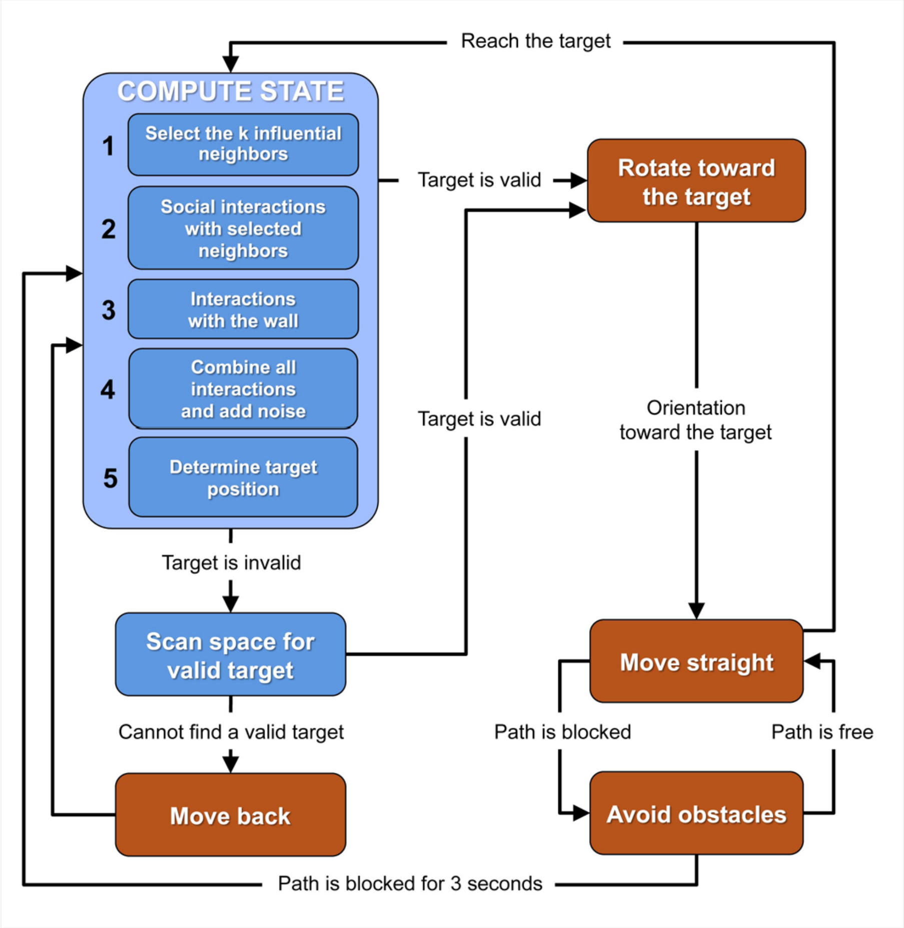

Fig. S8. Finite state machine diagram of one robot. *The decision-making processes of the robot (COMPUTE state) are show in blue. The movements of the robot (MOVE state) are shown in brown. In the COMPUTE state, the model determines a new target to reach by integrating the local information about the neighbors and the environment. A target is valid when this one is not blocked by the wall or the presence of neighbors. If the target is invalid, the computer tries to find a new target by the scanning method. If the scanning fails, the robot moves back 80mm and start again for model computing. If the decision target is valid, the robot switches into MOVE state, which includes three sub-states: Rotate, Move straight state and Avoid obstacle.*

The robot first rotates towards to the target and then, moves straight to it. If another running neighbor blocks the path, the robot uses a procedure to avoid the obstacles.

Reviewer #3:

I thank the authors for answering my previous concerns. I can recommend publication following some minor changes to the text (please see below).

#3.1. *Line 71 – Awkward sentence – please rephrase*

A: We modified the sentence as follows (lines 70-74):

> *"In golden shiners (Notemigonus crysoleucas), another work has sought to reconstruct the visual information available to each individual fish during collective evasion maneuvers [24]. In this species, it has been shown that the transmission of behavior in a school was best described by a model in which the response probability of a fish depends on the fraction of active neighbors perceived by that fish."*

#3.3. *Line 77 "In a previous work, we found experimental evidence that supports this assumption"*

A: Changed.

#3.4. *Line 81 ignore = "do not know"*

A: Changed.

#3.5. *Line 84 Awkward start to the sentence, please rephrase*

A: We modified the sentence as follows (lines 85-87):

> *"Once the influential neighbors of a focal fish have been identified, one must then understand how this individual integrates the information about the behavior of these neighbors."*

#3.6. *Line 107 – 112: This reads more like the start of a discussion and should be removed from the end of the introduction.*

A: Changed. These lines have been removed. The introduction ends at line 108.
