# Supplementary material for: Computational and robotic modeling reveal parsimonious combinations of interactions between individuals in schooling fish
Source: PLoS Comput Biol. 2020 Mar 16;16(3):e1007194. doi: 10.1371/journal.pcbi.1007194 (PMC7098660; doi:10.1371/journal.pcbi.1007194)
Supplement: Attachment — Submitted filename: Rebuttal_Letter.pdf [file pcbi.1007194.s026.pdf]

Thank you very much for submitting your manuscript "Computational and robotic modeling reveal parsimonious combinations of interactions between individuals in schooling fish" for consideration at PLOS Computational Biology. I considered the response to the previous review round satisfactory, and made a final review myself. We are likely to accept this manuscript for publication, providing that you modify the manuscript according to the following recommendations.
Indeed, I believe that the paper still presents some minor issues that needs to be addressed before publication.

We thank the Editor for his positive assessment of our revised manuscript and address his remarks below, which have been separated in 5 points.

1. The major findings of the paper should be spelled out more clearly. At the current stage, the reader may get confused about what strategy is the best, and what value of k. To the best of my understanding, all strategies work well for k=2, leading to a quantitative match between experiments and simulations, but only the "most influential" strategy presents a behaviour qualitatively similar to the fish data for k=1. The robot experiments strengthen this aspect, providing a better (but not exact) quantitative match with the fish experiments for k=1. The way in which this message is passed in the current version is biased in favour of the k=1 match, which is mostly qualitative, providing in my opinion a spurious message. It actually seems that the best strategy to explain the data is "most influential" with k=1, but this is not true. Rather, k=1 most-influential neighbours is a sufficient number to get qualitative match, but k=2 neighbours are necessary to get a more quantitative match. I think that the abstract, summary, results and conclusions should be slightly rephrased.

Answer: First, we never stated in the text that k=1 MOST INFLUENTIAL is the best strategy to explain the fish data. Moreover, it should be clear, as repeatedly expressed in the text, that the main purpose of this work is to explore and compare different interaction strategies.

As discussed in the introduction and the discussion, several studies (not only in fish) have found that individuals can effectively interact with very few neighbors (sometimes as few as k=1 or 2). Our conclusion is that the MOST INFLUENTIAL strategy is overall the best strategy compared to the two others *for a given* k=1 or 2 (for k>2, all strategies are essentially comparable, as explained in the text) and is the only one leading to cohesive groups for large number of agents N. For the model in particular, we start the discussion by stating explicitly that considering 2 neighbors improve the agreement fish experiments for all strategies and that all strategies give satisfactory results. Finally, we also argue that the notion of MOST INFLUENTIAL neighbors could be biologically relevant (see our improved discussion following your point **4**).

More precisely, in the **Results** section, supporting these conclusions, we have shown that

a. k=1 only leads to cohesive (and quite polarized) groups for the MOST INFLUENTIAL strategy, in the model and in the robotic platform. In addition, our results for large groups in an unbounded model clearly shows that the NEAREST strategy fails completely at forming cohesive groups unless one considers k at least of order N/2 (which should be unrealistic for large N). On the contrary, k=1 (and presumably

higher k even more; see our reply to your point **3**) already leads to coherent groups for the MOST INFLUENTIAL strategy. In relation to cognitive overload (see point **4** below), it is quite satisfying that only considering one neighbor (but, the most influential one) can already lead to cohesive and polarized groups.

b. Yet, in the discussion of the results of the model, we have clearly stated that the results for all strategies improve with k, and that fork=2, the NEAREST strategy is essentially as "good" (allow us this shortcut in this short discussion) as the MOST INFLUENTIAL strategy (albeit with a clearly weaker cohesion; see Table 1), while the RANDOM strategy remains significantly "worse" (cohesion and polarization).

c. In the robotic platform, the MOST INFLUENTIAL strategy is still significantly "better" than the two other strategies for k=2 (see Table 2). In fact, the MOST INFLUENTIAL strategy for k=1, is still "better" than the NEAREST strategy for k=2.

d. Hence, for the model and robotic platform, k=2 MOST INFLUENTIAL is "better" than k=1 MOST INFLUENTIAL (as expected; see the discussion of your point **3** below), but the important message is that it is "much better" than the two other strategies for k=1, and overall still better for k=2.

We have carefully reread the summary and our discussion of the results (Results and Discussion sections), and could not honestly identify the misrepresentation of our results that the Editor is alluding too.

Yet, in the abstract, we agree that the sentence "*We find that groups of robots are able to reproduce the behavioral patterns observed in groups of five fish when each robot only interacts with its neighbor having the strongest effect on its heading variation, whereas increasing the number of interacting neighbors does not significantly improve the group coordination.*" could be misleading. Hence, we have rewritten this sentence to focus on the result (c) summarized above, and we have added a short sentence announcing our discussion of the biological relevance of the notion of "most influential neighbors".

2. I see that the data about k=3 for the MOST INFLUENTIAL strategy with robots are missing, and I couldn't find in the main text an explanation for this. This is a shortfall that must be clearly explained.

Answer: Indeed, the experiment for k=3 and MOST INFLUENTIAL was not performed, which is now explicitly stated early in the manuscript in Line 452. This was clearly a mistake but which cannot be corrected in the near future as our colleague managing and performing this experiment has returned to China.

3. The rationale for choosing k=1 in the simulation about "collective motion of larger groups in an unbounded domain" should be clearly spelled out. I would have expected simulations also for k=2, which could show improved polarisation even for large groups. Although the behaviour resulting from k=1 is described sufficiently, the fact that with k=2 the same pattern arises is not guaranteed. Why no data has been included about k=2 for large groups?

Answer: these additional simulations were requested by a referee in order to confirm that the surprising result of the occurrence of cohesive groups in a bounded domain already for k=1 and only for the MOST INFLUENTIAL strategy was not a mere artifact of the confinement. In principle, adding a second neighbor can only strengthen cohesion and polarization, as already found in a bounded domain for the fish model and the robotic platform. We sincerely believe that, at this stage, a further study of the model for large schools in an unbounded domain is well beyond the present work, as it calls for addressing many other issues. In particular, in addition to the question of the properties of a large model school as a function of k, we plan to study in the near future the conditions of the occurrence of the swarming, milling/vortex, and polarized schooling collective phases when varying the parameters of the model: k/strategy; and most importantly, the intensity and range of the attraction and alignment interactions (in the spirit of [32], which required hundreds of hours of numerical simulations). Note that we recently found in [15] that the range of interactions (attraction an alignment) for *Danio rerio* (zebrafish) is much shorter than for *Hemigrammus rhodostomus*, explaining the weaker social behavior of the former (group cohesion and polarization).

4. In the conclusions, lines 651-663 are disconnected from the previous text, and should be better introduced. This paragraph has been included to motivate the biological plausibility of the proposed strategies. The important information is that identification of the most relevant neighbours does not require an explicit consideration of all the neighbours, which would be cognitively too demanding. It is suggested that this process is carried out in parallel by the forebrain, hence providing a cognitively plausible explanation of a strategy that would otherwise be very demanding. This should be clarified, possibly also discussing that the most salient stimuli could well be filtered in a way to automatically determine those that are most salient for the movement decision, i.e., suggesting a mechanisms for the computation of mostly influential individuals. This would somehow address the self-referentiality problem previously mentioned by the reviewers.

Answer: First, we agree that a transition was missing at the beginning of this paragraph. In addition, since the question of the computational complexity on a computer and in the biological context of the MOST INFLUENTIAL strategy was also raised by the referees, we have added a paragraph (Line 652) about the purely computational complexity (on a computer) of the MOST INFLUENTIAL strategy, followed by a modified and extended paragraph addressing its possible biological relevance. We hope that the Editor will find that these two paragraphs now discuss this issue adequately.

5. Minor issues:
lines 106-107: you may also mention collision avoidance
line 146: radius -> radii
lines 177-178: make a new sentence out of the brackets, or a footnote

Answer: Done
